# A versatile 5′ RACE-Seq methodology for the accurate identification of the 5′ termini of mRNAs

**DOI:** 10.1186/s12864-022-08386-y

**Published:** 2022-02-26

**Authors:** Panagiotis G. Adamopoulos, Panagiotis Tsiakanikas, Irene Stolidi, Andreas Scorilas

**Affiliations:** Department of Biochemistry and Molecular Biology, National and Kapodistrian University of Athens, Panepistimiopolis, 15701 Athens, Greece

**Keywords:** Rapid amplification of cDNA ends, Nanopore sequencing, Massive parallel sequencing, 5′ cap, 5′ RACE, UTRs

## Abstract

**Background:**

Technological advancements in the era of massive parallel sequencing have enabled the functional dissection of the human transcriptome. However, 5′ ends of mRNAs are significantly underrepresented in these datasets, hindering the efficient analysis of the complex human transcriptome. The implementation of the template-switching mechanism at the reverse transcription stage along with 5′ rapid amplification of cDNA ends (RACE) constitutes the most prominent and efficient strategy to specify the actual 5′ ends of cDNAs. In the current study, we developed a 5′ RACE-seq method by coupling a custom template-switching and 5′ RACE assay with targeted nanopore sequencing, to accurately unveil 5′ termini of mRNA targets.

**Results:**

The optimization of the described 5′ RACE-seq method was accomplished using the human *BCL2L12* as control gene. We unveiled that the selection of hybrid DNA/RNA template-switching oligonucleotides as well as the complete separation of the cDNA extension incubation from the template-switching process, significantly increase the overall efficiency of the downstream 5′ RACE. Collectively, our results support the existence of two distinct 5′ termini for *BCL2L12*, being in complete accordance with the results derived from both direct RNA and PCR-cDNA sequencing approaches from Oxford Nanopore Technologies. As proof of concept, we implemented the described 5′ RACE-seq methodology to investigate the 5′ UTRs of several kallikrein-related peptidases (*KLKs*) gene family members. Our results confirmed the existence of multiple annotated 5′ UTRs of the human *KLK* gene family members, but also identified novel, previously uncharacterized ones.

**Conclusions:**

In this work we present an *in-house* developed 5′ RACE-seq method, based on the template-switching mechanism and targeted nanopore sequencing. This approach enables the broad and in-depth study of 5′ UTRs of any mRNA of interest, by offering a tremendous sequencing depth, while significantly reducing the cost-per reaction compared to commercially available kits.

**Supplementary Information:**

The online version contains supplementary material available at 10.1186/s12864-022-08386-y.

## Background

Rapid amplification of cDNA ends (RACE), also described as “one-sided” PCR or “anchored” PCR, is a molecular technique that enables the amplification of nucleic acid sequences from a messenger RNA (mRNA), between a specific internal region and either the 3′ or the 5′ end of the mRNA. Since its establishment in 1988, RACE has emerged as the main strategy used to determine both the 5′ and/or the 3′ untranslated regions (UTRs) of any mRNA transcript, defining the transcription start point(s) as well as the poly(A) tail sites, accordingly [1–3]. The 3′ RACE is a well-described and optimized methodology, which exploits the natural poly(A) tail of mRNAs as a generic priming site for PCR amplification and takes place in two distinct steps. In the first step, mRNAs are reversely transcribed into cDNA, using a reverse transcriptase and an oligo-dT adapter as primer. The second step involves a PCR amplification using a gene-specific primer (GSP) that anneals to a region of a known exon sequence and a universal primer that is designed to anneal to the adapter sequence that was used in the previous step of the reverse transcription. This approach enables the identification of any unknown mRNA sequence located between this specific exon and the poly(A) tail [4].

Although similar approaches have also been employed for the characterization of the 5′ UTRs, 5′ RACE is often a more complicated and challenging procedure, since the 5′ ends of mRNAs lack any generic priming sites. As a result, the major challenge of 5′ RACE compared to the 3′ RACE is the accurate incorporation of an adapter sequence in the 5′ end of the mRNAs that will lead to the unbiased characterization of 5′ UTRs. The successful identification of novel 5′ UTRs is of high significance, since these regions are firmly associated with mRNA stability, the subcellular localization and/or the translational efficiency of mRNAs [5]. Initial attempts included the use of homopolymeric tailing or ligation anchored tailing [6]. In homopolymeric tailing approaches a GSP primer is used to generate first-strand synthesis products. Thereafter, a poly(A) tail is incorporated in cDNA products, using terminal deoxynucleotidyl transferase (TdT) and dATP. The cDNA products are amplified using a poly(T) adapter along with the first GSP primer that anneals upstream compared to GSP used in reverse transcription. Finally, a consecutive nested PCR, using a universal primer that anneals within the poly(T) adapter sequence alongside a second GSP, is employed to further increase the yield of 5′ end-specific PCR products [5]. Although this is a straightforward approach, it usually generates 5′ partial cDNA clones of a specific gene of interest, rendering the simultaneous study of 5′ UTRs in multiple genes extremely time-consuming. To mitigate this limitation and enhance the specificity of the 5′ RACE, later approaches intended for the acquisition of full-length 5′ cDNAs. A common characteristic shared by these methodologies is the inclusion of an anchored priming site to the 5′ end of the mRNA and cDNA, before or during RT, respectively. The inclusion of an anchor primer to the unknown 5′ ends of the mRNAs requires the removal of cap structure through various enzymatic steps, using tobacco acid pyrophosphatase (TAP) [7].

However, these approaches involved various enzymatic reactions after the synthesis of the first-strand cDNA molecules, thus leading to the production of cDNA samples with low integrity. In an effort to circumvent the significant limitations of this framework, a more simple and efficient strategy was introduced by Clontech in 1996, broadly known as SMART™ technology [8]. This technology was designed to take advantage of terminal transferase activity of moloney murine leukemia virus (MMLV) reverse transcriptase during first strand cDNA synthesis. In detail, a few non-templated cytosines are added in a 5′-cap-dependent manner as a final step during reverse transcription (RT) of mRNAs [9]. Several variations of the described methodologies can be found in commercially available, ready-to-use kits provided by leading biotech companies. However, in most cases such kits provide a limited number of reactions usually for an immense price, dramatically increasing the cost per reaction.

Technological advancements in the era of massive parallel sequencing and especially RNA sequencing (RNA-seq) enabled the meticulous investigation of alternative splicing (AS) mechanism, which is the key of protein diversification [10]. However, the prevalence of AS events within 5′ UTRs or the existence of alternative transcription initiation sites have been overlooked [11]. These issues were greatly resolved by the fifth edition of the Functional Annotation Mammalian Genome (FANTOM5) scientific consortium, which provided a functional annotation of the human genome by coupling CAGE (Cap Analysis of Gene Expression) with high-throughput sequencing [12–14]. Although FANTOM5 provided a summarizing overview of the transcription start sites (TSSs) distribution in a genome-wide scale by analyzing approximately 400 distinct cell types, several structural variations of 5′ UTRs remained undetected, thus critically limiting our understanding concerning the impact of 5′ UTR diversification on the regulation of gene expression [15, 16]. This fact can be mainly attributed to the lack of both sequence depth and coverage, reflecting the intrinsic limitations of both CAGE application and the downstream analytical processing of the results. As an alternative, a RACE-seq methodology which combines massive parallel sequencing with 5′ RACE, had already been established since 2009 and attempted to eliminate this gap of information, providing a better overview of the 5′ and 3′ UTRs [17]. The characterization of RACE products with high-throughput sequencing approaches is time-efficient, ultra-sensitive, less costly and technically feasible compared to CAGE or traditional characterization of RACE products that involve molecular cloning followed by Sanger sequencing of specific clones. The ongoing refinement of RACE-seq methodologies will ameliorate the structural characterization of non-coding RNAs (ncRNAs) as well as the accurate determination of UTR boundaries or the existence of alternative TSSs [18, 19].

In the current study, we developed a custom assay for the implementation of targeted 5′ RACE-seq methodology, combining an *in-house* developed 5′ RACE protocol with massive parallel sequencing to fully elucidate the 5′ mRNA termini of several genes, using an established panel of 51 human cell lines. For this purpose, we designed template-switching oligos (TSOs) with distinct features and optimized several conditions of the RT reaction. Both the efficiency and validity of the presented 5′ RACE-seq approach were evaluated, using as control gene the extensively studied, apoptosis-related *BCL2L12* gene [20]. Then, as proof of concept, we applied our method to investigate 5′ UTRs of the human kallikrein-related peptidases (*KLKs*) gene family members. The obtained 5′ RACE amplicons were subjected to massive parallel sequencing to accurately determine their 5′ ends, while our results were further validated using publicly available datasets from the Sequence Read Archive (SRA). Overall, our kit-free approach exploits all the advantages of the template-switching mechanism and at the same time reduces the cost of the 5′ RACE implementation, whereas the optimization of the first-strand cDNA synthesis protocol further increases the efficiency of the method. As a result, this assay can be used coupled with massive parallel sequencing approaches as the method of choice for the identification of alternative transcription start sites and 5′ UTRs.

## Results

### Optimization of a custom designed RT protocol

To investigate how the chemical composition and/or the specific modifications of different TSOs affect the overall performance of the RT reaction, we used three TSOs with distinct properties, as shown in Table 1. All three TSOs were employed both in a typical 1-step RT protocol and in our custom designed 2-step RT assay, using a common RNA template, which led to the production of six distinct cDNA samples. The diverse effect of the various optimizations during RT procedure was assessed using the extensively studied 5′ UTR of the *BCL2L12* gene. In detail, a nested 5′ RACE assay was employed targeting the 5′ UTR of the *BCL2L12* gene, using two universal primers designed to anneal at the TSO sequence along with two GSPs (Supplementary Table 1). Finally, the derived nested RACE products were electrophoresed for the assessment of the results.

**Table 1:** Template-switching oligonucleotides (TSOs) and 5′ RACE primers used in the present work

Name	Sequence (5′ → 3′)	Description
dTSO	AAGCAGTGGTATCAACGCAGAGTACGCGGG	TSO with typical DNA oligonucleotides
drTSO	AAGCAGTGGTATCAACGCAGAGTACGCr(GGG)	Chimeric DNA/RNA oligo sequence that carries 3 riboguanosines r(GGG) at its 3′ end
C3-TSO	AAGCAGTGGTATCAACGCAGAGTACGCr(GGG)-C3	Chimeric DNA/RNA oligo sequence that carries 3 riboguanosines r(GGG) at its 3′ end and a 3′ C3-spacer modification
F	AAGCAGTGGTATCAACGCAGAG	Forward universal primer designed to anneal at the TSO sequence for RACE reaction
Fnested	CAGTGGTATCAACGCAGAGTAC	Forward universal primer designed to anneal at the TSO sequence for nested RACE reaction

**Fig. 1:**
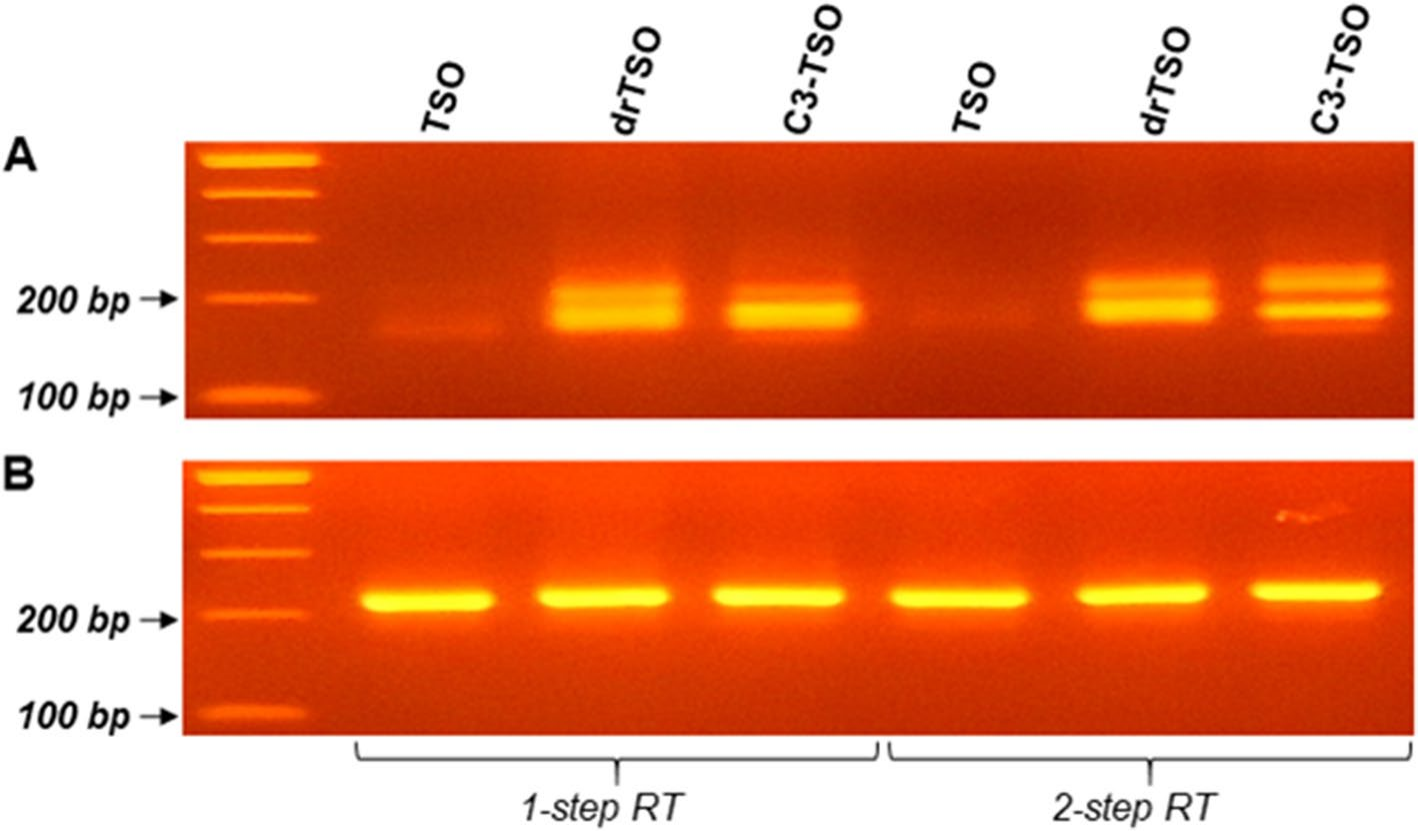
Electrophoresis results of the amplicons derived from nested 5′ RACE and the housekeeping gene amplification. **A** Electrophoresis of nested 5′ RACE amplicons corresponding to the 5′ UTR of the human *BCL2L12* gene, using the three distinct TSOs described in the present study. Each TSO was incorporated both in a typical 1-step and a custom designed 2-step RT reactions before the implementation of the nested 5′ RACE. **B** Electrophoresis of the amplicons corresponding to the *GAPDH* mRNA amplification, which was used as housekeeping for the present work

The electrophoresis results clearly demonstrate that a DNA/RNA hybrid TSO (drTSO) is superior in terms of overall performance than a TSO composed of typical DNA nucleotides, which is in accordance with findings of previous studies [21]. Although DNA/DNA interaction is generally considered to be stable, the incorporation of ribonucleotides (specifically rG) at the 3′ end of the TSO emerges as a key element that significantly increases the efficiency of the template-switching mechanism and thus the overall amount of the produced full-length cDNAs. This is strongly supported by our results, since the incorporation of the non-hybrid TSO during RT failed to produce full-length cDNAs with intact 5′-ends, thus resulting in a negative 5′ RACE result. These findings were observed in both RT protocols that were applied (Fig. 1).

Furthermore, as shown by the obtained results, although drTSO produces notably higher yields of the specific amplicon that corresponds to the 5′ UTR of *BCL2L12* gene, leading to non-specific hybridization events within random internal sequences of RNA, thus acting as an internal RT primer and subsequently leading to the creation of RT byproducts. In addition, although the developed 2-step RT approach led to a significant byproduct decrease as compared to the typical 1-step RT protocol, it still failed to eliminate all random byproducts, as spurious bands still appeared in the electrophoresis gel (Fig. 1).

To significantly decrease the presence of non-specific products obtained during RT reaction we used the same DNA/RNA hybrid TSO that also incorporated a 3′ C3-spacer modification (C3-TSO). Since 3′ C3-spacer is a versatile modification in which a 3-carbon chain (C3) is attached to the terminal 3′ hydroxyl group, this modification in the 3′ end of the TSO would be expected to act as a blockage, preventing the extension of non-specific products during first-strand cDNA synthesis. As expected, our results confirmed that notion, since C3-TSO enabled the production of specific full-length cDNAs in terms of 5′-ends, thus eliminating the vast majority of byproducts, but leading to a notable decrease of the 5′ RACE product yield, as compared to the unmodified drTSO (Fig. 1).

Finally, we observed that the addition of TSO along with an excess of RT enzyme at a specific point during RT, in which the first-strand cDNA synthesis would be theoretically completed for most mRNAs, could greatly improve the transition from RNA template to the TSO sequence, thus further increasing the efficient production of full-length cDNAs. Of note, this modification to the protocol critically facilitates the successful full-length cDNA production of large transcripts (> 13 kb), in which the accurate determination of their 5′ ends remains a challenging process.

### Nanopore sequencing unveils the primary and alternative 5′ ends of *BCL2L12* gene

**Fig. 2:**
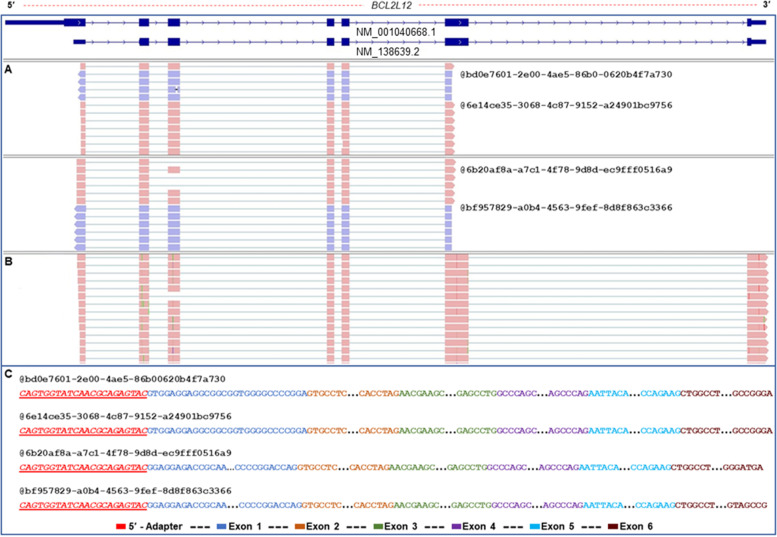
Comparative analysis of aligned sequencing reads on the human *BCL2L12* gene, derived from the presented 5′ RACE-seq approach and publicly available Direct RNA sequencing datasets. **A** Visualization of the aligned sequencing reads derived from the presented 5′ RACE-seq approach. **B** Visualization of the aligned sequencing reads derived from publicly available Direct RNA sequencing datasets deposited on the Sequence Read Archive database (SRA). **C** Indicative raw nanopore sequencing reads from the generated FASTQ file of the presented 5′ RACE-seq approach, highlighting the incorporation of the drTSO specifically to the 5′ end of each target mRNA transcript

Nanopore sequencing of the nested 5′ RACE products, derived from both drTSO and C3-TSO, enabled the identification of novel 5′ UTRs of *BCL2L12*. Of note, neither of our findings confirms the existence of the two distinct in silico curated 5′ UTRs for *BCL2L12* that are presented by GenBank® (Fig. 2). Instead, our data clearly supports the existence of two distinct 5′ termini sites located a few nucleotides (nt) downstream of the annotated 5′ UTR termini of the main *BCL2L12* mRNA transcript, *BCL2L12* v.1 (Supplementary Fig. 1). The detected UTRs of *BCL2L12* demonstrated a wide expression pattern in cDNAs originating from multiple human tissues (Supplementary Fig. 2). Hence, our data only support the existence of the annotated translation initiation codon that resides on the second exon of *BCL2L12* v.1 (Fig. 2).

To further evaluate the validity of our approach, we compared our results with publicly available datasets from direct RNA sequencing (Sequence Reads Archive database, SRA) and the FANTOM5 project. It should be mentioned that direct RNA approach enables the sequencing of the full-length mRNA molecules, without involving the template-switching mechanism. Despite this fundamental difference between our targeted RACE-seq approach and direct RNA sequencing, both methodologies are in complete accordance regarding the 5′ UTR of the human *BCL2L12* gene, since they support the same 5′ termini sites (Fig. 2). Finally, our results do not support the existence of several TSS peaks as they presented in FANTOM5 for *BCL2L12* (Supplementary Fig. 3).

**Fig. 3:**
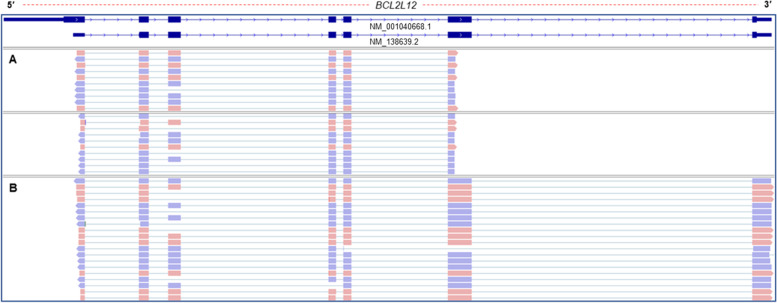
Comparative analysis of aligned sequencing reads on the human *BCL2L12* gene, derived from the presented 5′ RACE-seq approach and an implemented nanopore PCR-cDNA Sequencing methodology. **A** Visualization of the aligned sequencing reads derived from the presented 5′ RACE-seq approach. **B** Visualization of the aligned sequencing reads derived from PCR-cDNA Sequencing methodology (SQK-PCS109, ONT)

Additionally, since PCR-cDNA sequencing approach from Oxford Nanopore Technologies (ONT) enables the identification of the 5′ mRNA ends based on a template-switching mechanism and the amplification of the full-length cDNAs, this approach is ideal for comparison of the 5′ mRNA ends that derived from the presented approach. For this purpose, we employed the PCR-cDNA sequencing approach on the same biological material that was used for targeted 5′ RACE-seq, to further evaluate the validity of our results. Bioinformatics analysis of the PCR-cDNA sequencing datasets confirmed that both approaches are completely in line regarding the detected 5′ mRNA ends of *BCL2L12*, however, the presented methodology provided a notably increased sequencing depth and coverage (Fig. 3).

### Validation of annotated 5′ UTRs of the *KLK* gene family members

**Fig. 4:**
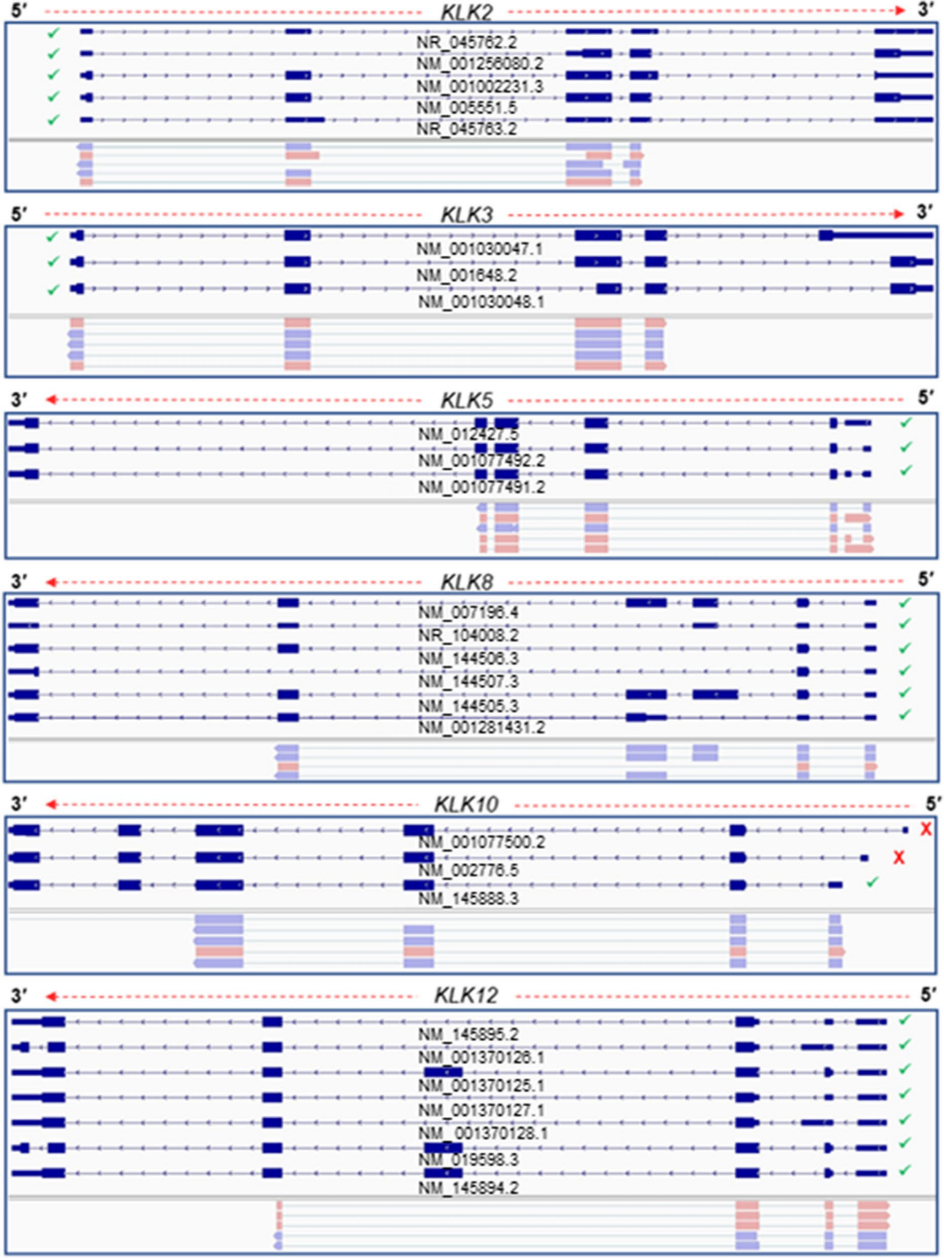
Visualization of aligned sequencing reads, derived from our custom 5′ RACE-seq approach, which confirm the existence of annotated UTRs for several members of the human *KLK* gene family. The human genome hg38 (GRCh38) was used as reference for the alignment process with minimap2

As proof of concept, we implemented the described 5′ RACE methodology to investigate the 5′ UTRs of several human *KLK* gene family members. Thereby, we selectively amplified 5′-ends of several human *KLK* gene family members, using drTSO and GSPs for each *KLK* mRNA target. The produced amplicons were then subjected to nanopore sequencing as described in Materials and Methods. Summarizing our results, we validated annotated 5′ UTRs in *KLK2*, *KLK3*, *KLK5*, *KLK8*, *KLK10* and *KLK12* (Fig. 4). Transcript variants of *KLK2*, *KLK3*, *KLK8* and *KLK12* are characterized by a single annotated 5′ UTR, which has been confirmed by the presented approach. Additionally, all three distinct annotated 5′ UTRs of *KLK5* were confirmed. On the contrary, *KLK10* is characterized by three annotated 5′ UTRs, however our results validated one UTR. In specific, sequencing results confirmed only the 5′ UTR that corresponds to *KLK10* v.2 (GenBank® accession number: NM_145888.3), whereas the 5′ UTRs of *KLK10* v.1 and v.3 (GenBank® accession numbers: NM_002776.5 and NM_001077500.2, accordingly) were not detected (Fig. 4).

### Identification of novel 5′ UTRs of the *KLK* gene family members

**Fig. 5:**
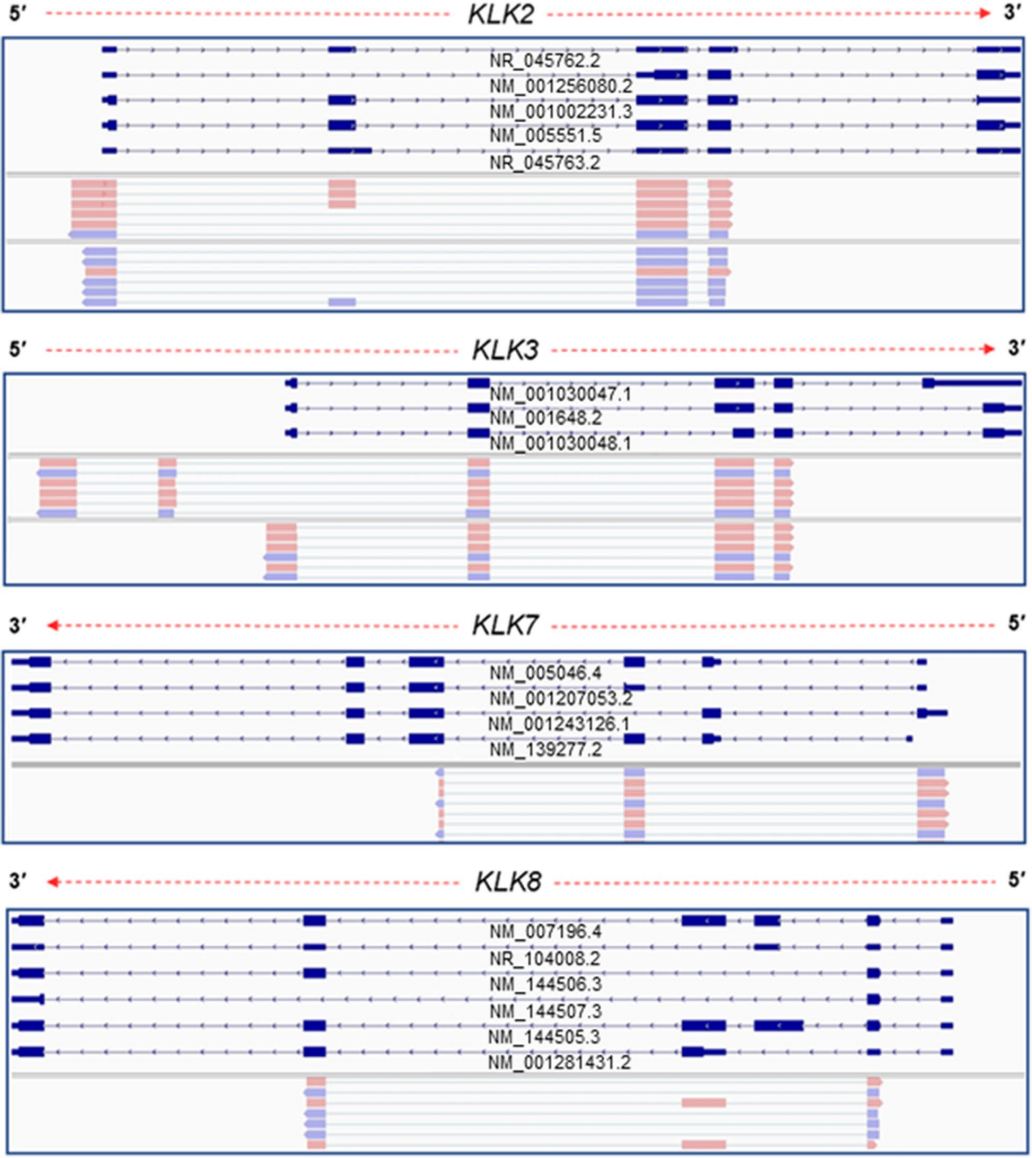
Visualization of aligned sequencing reads from the presented 5′ RACE-seq approach, which confirm the existence of previously uncharacterized 5′ UTRs for several members of the human *KLK* gene family. The human genome hg38 (GRCh38) was used as reference for the alignment process with minimap2

Interestingly, besides the confirmation of the annotated 5′ UTRs, our results elucidated multiple previously unknown 5′ UTRs of *KLK2*, *KLK3*, *KLK7* and *KLK8* (Fig. 5). In detail, we identified 2 novel 5′ UTRs that are extended compared to the annotated 5′ end of *KLK2* v.1 (GenBank® accession number: NM_005551.5). Of note, the novel 5′ UTRs are observed in similar expression levels to the annotated 5′ UTR, thus they most likely represent highly abundant findings. Similarly, in *KLK3* our analysis revealed a notably abundant, novel 5′ UTR, which is located upstream of the annotated one. Interestingly, we observed a novel 5′ UTR of *KLK3* with an entirely different 5′ boundary architecture, which is formed by the consecutive splicing of two novel exons (Supplementary Fig. 1). Concerning *KLK7*, although the 5′ boundary of all the sequencing reads represents the one of the annotated *KLK7* v.4 (GenBank® accession number: NM_001243126.1), the structure of 5′ UTR that was observed in our data constitutes a combination of splicing events between *KLK7* v.3 and v.4 (Fig. 5). Finally, in most of the sequencing reads that correspond to *KLK8*, we observed a 5′ end truncation, which results to a different and uncharacterized 5′ UTR (Supplementary Fig. 1).

## Discussion

It is well documented that the 5′ UTR architecture is crucial for both mRNA stability and translational efficiency [22]. The functional annotation of the human genome regarding the TSSs has been conducted within the framework of FANTOM5 research consortium. Although FANTOM5 project identified at the nucleotide-level resolution the most prominent TSSs across the human genome, their association with the downstream gene structures was neglected [23]. This limitation arises from the fact that FANTOM5 project is based on extremely short sequencing reads (~ 100 nt) for the identification of TSSs and therefore fails to identify downstream mRNA sequences. Alternatively, the determination of the 5′ UTRs of protein-coding genes, can be accomplished using 5′ RACE methodology [24]. The initial 5′ RACE efforts were characterized by several drawbacks, such as the requirement of an excess of initial RNA material as well as the conduction of multiple enzymatic procedures, making the entire process extremely time-consuming [7, 25–27]. Later, the development of a 5′ CAP-sensitive reverse transcription that exploits the intrinsic terminal transferase activity of MMLV reverse transcriptase, which adds a few non-templated cytosines at the 3′-end of the produced cDNA reduced, to some extent, the undesired limitations. In this approach, also known as template-switching, a generic adapter oligonucleotide sequence is used to efficiently anneal at the non-templated nucleotides of the cDNA [9].

In the current study, we developed a cost-effective 5′ RACE-seq assay, exploiting the methodological aspects of the template-switching mechanism. Nevertheless, we incorporated several key modifications to further increase the efficiency of the traditional approach as well as the overall accuracy of the obtained 5′ RACE amplicons. To the best of our knowledge, the optimization of mRNAs’ reverse transcription constitutes the crucial step to achieve the successful recovery of full-length cDNAs [6, 28]. Conventional full-length cloning techniques provide 5′ truncated cDNAs ends that are mainly caused by premature termination of reverse transcription or during blunt-end generation process [29]. Of note, the efficiency of any described 5′ RACE protocol significantly relies on the RT step and more particularly the choice of the reverse transcriptase that will be used. During the synthesis of the first-strand cDNA, the stable secondary structures that may exist throughout the sequence of the mRNAs may cause early termination of the generated cDNA molecules and therefore the creation of “full-length” cDNA libraries with enriched 5′ cap sites is challenging. Consequently, higher temperatures of the RT reaction are expected to lead to fewer sites with stable secondary structures and as a result reverse transcriptases with high optimal temperatures for cDNA synthesis are recommended for this purpose. In the present study, we used the SMARTScribe™ Reverse Transcriptase, which possesses an optimal temperature for cDNA synthesis at 42 °C and demonstrated efficient results in terms of generated cDNAs with enriched 5′ cap sites. Although, the described 5′ RACE-seq method was conducted with SMARTScribe™ Reverse Transcriptase, our results have shown that it can also be efficiently implemented with other reverse transcriptases with similar optimal temperatures and with terminal deoxynucleotidyl transferase (TdT) activity, such as Maxima H Minus Reverse Transcriptase.

Taking into consideration all the afore-mentioned criteria, we used a custom oligo-dT adapter as RT primer, designed to anneal at the poly(A) tail of the mRNAs, along with a TSO in the same RT reaction mix to specify the actual 5′- and 3′-ends for the total amount of mRNAs that are transcribed. The produced cDNA can be used as initial material to study both 5′ and 3′ boundaries of multiple targets, hence eliminating the need for an increased amount of starting RNA material and/or the conduction of multiple, time-consuming RT reactions, separately for each target gene, as it has been described in previous approaches [29, 30].

The use of a TSO that efficiently anneals at the 5′ ends of cDNAs has emerged as one of the most important parameters to optimize in the described methodology. We evaluated three distinct TSOs, same in terms of nucleotide sequence, but different regarding their monomeric unit composition and the presence of chemically modified nucleotides. The first TSO was composed entirely of DNA nucleotides and failed to efficiently anchor to the 5′ ends of human mRNAs (Fig. 1). Although the incorporation of such TSOs is questionable for the study of 5′ ends in vertebrates, due to their increased complexity, it can still provide robust results in case of some organisms [31]. Instead, both efficiency and overall accuracy of the 5′ RACE assay were dramatically increased when we used DNA/RNA hybrid TSOs. These hybrid oligos are characterized by the presence of three consecutive riboguanosines in their 3′-end (rGrGrG-3′). As it was expected, the presence of rG is an essential prerequisite to enable the highest template-switching efficiency during the RT reaction [21, 32]. Our experimental design was further refined through the development of a hybrid TSO, bearing a chemically modified rG nucleotide in its last 3′ position, by adding a C3-spacer as blocking strategy. The rationale behind this decision is related to potential misspriming events that may occur during RT reaction between the unblocked DNA/RNA TSO and the mRNA template, resulting in an increase of artifactual cDNA products. This is a common issue when RACE methodology is applied, therefore several strategies have been developed to improve the fidelity of the obtained results [5, 33–35]. The qualitative evaluation of the presented methodology was conducted using *BCL2L12* as control gene. To produce 5′ RACE amplicons, we used the three different TSOs presented in this study (Table 1). We observed that the use of both hybrid TSOs led to the production of two distinct 5′ RACE amplicons for *BCL2L12*, which represent its two different 5′ ends (Fig. 1). The derived electrophoresis results come in accordance with the aligned sequencing data obtained from both Direct RNA and PCR-cDNA sequencing methodologies from Oxford Nanopore Technologies (Figs. 2 & 3). Furthermore, we observed a notable discrepancy in both efficiency and specificity between the 5′ RACE amplicons obtained by different hybrid TSOs. In detail, the utilization of the blocked hybrid TSO significantly increases the specificity, however it slightly reduces the overall yield of the derived *BCL2L12* 5′ RACE products. On the contrary, the use of the unblocked TSO provides 5′ RACE products of *BCL2L12* with high yield although lacking specificity due to the increased amount of 5′ RACE byproducts (Fig. 1). This is a key observation, as it can determine the selection of the most appropriate TSO depending on the intrinsic characteristics of target mRNA transcripts that are studied. The use of the blocked TSO provides reliable results and it is strongly recommended when the expression levels of the target gene are high, whereas the use of the unblocked TSO could benefit the study of low expression genes by increasing the overall yield of the 5′ RACE amplicons. However, the implementation of nested PCR to produce 5′ RACE products made the quantitative evaluation of the different TSOs not feasible.

Another crucial parameter that was evaluated in the context of the current study, was the timepoint of the TSO addition to the RT reaction mix. We observed that the complete separation of the cDNA extension incubation from the template-switching process, by adding the TSO in a distinct second step during RT reaction, increases the overall yield and accuracy of the derived 5′ RACE products (Fig. 1). Of note, a recent study reported that the addition of non-templated nucleotides by RT enzyme and template-switching occur concurrently, while their sequential separation did not alter the overall efficiency of template switching [32]. However, in our approach TSO was included to RT reaction with a small amount of additional RT enzyme. Although, the reasons for the observed result are not yet completely understood, the simultaneous addition of both TSO and RT enzyme improves the efficiency of the template-switching mechanism.

The described parameters were optimized in a cDNA pool derived from an established panel of 51 cell lines corresponding to 16 primary tissues for the apoptotic *BCL2L12* gene and subsequently implemented in *KLK* gene family members, which exhibit differential expression patterns in human tissues and at the same time present a plethora of transcript variants resulting from multiple splicing events within their pre-mRNAs. Although the 5′ UTRs of the *KLK* gene family members are poorly investigated, *BCL2L12* has been extensively studied recently, rendering *BCL2L12* as an excellent control gene to optimize and evaluate the results of the presented approach. Our results indicated two truncated 5′ UTRs compared to the initially described 5′ end of the main mRNA transcript, as the most prevalent in our established panel of 51 human cell lines. These results are in accordance with recent studies that report a significant shorter 5′ UTR for *BCL2L12*. Although the upstream limits of the identified 5′ UTRs are not identical to the one reported in the previous study, they span within the region of 345 and 752 nt after the initially characterized transcription-start site, which has been found to present the highest promoter activity [20, 36]. Similar results are also observed after the analysis of publicly available nanopore Direct RNA and *in-house* generated PCR-cDNA sequencing datasets, thus enhancing the validity of our approach (Fig. 2). It should be mentioned that long-read direct RNA sequencing provided by nanopore platforms exploits a different approach compared to template-switching mechanism to unveil complexity of 5′-ends of mRNAs [37].

However, the interpretation of the obtained results should be conducted meticulously, under the prism of the promiscuity characterizes transcription machinery as well as the downstream repression mechanisms that limit potentially atypical transcript variants such as nonsense-mediated decay NMD [38]. Moreover, it is well established in the literature that genetic alterations of cancer cells lead to an excessive and dysregulated transcription [39]. In this study we used exclusively cancer cell lines as biological material to study the 5′ ends of the transcript variants for the selected target genes. Due to this limitation, the future expansion of this study to additional cell lines including non-cancerous cellular states is necessary to establish whether the described TSSs are employed in a cell and/or tissue specific manner or exhibit generalized expression patterns. Finally, in the current study two GSPs were designed to produce 5′ RACE amplicons for the target genes. Consequently, it should be mentioned that the presented results may include only a subset of the transcript variants that encompass the identified TSS. Despite the fact that even a single GSP can lead to 5′ RACE amplicons that offer valuable information regarding the connection of TSS and downstream gene structure, the use of more GSPs for a specific gene is highly recommended or even necessary to provide a detailed overview of link between different TSS and the structure of individual transcript variants.

## Conclusion

In summary, in this work we present a custom 5′ RACE-seq methodology, based on the template-switching mechanism and targeted nanopore sequencing. The described approach enables the broad study of 5′ UTRs, offers a tremendous sequencing depth due to the targeted amplification of the 5′ ends and at the same time significantly reduces the cost-per reaction compared to commercially available kits. Our 5′ RACE-seq approach was optimized on the extensively studied human *BCL2L12* gene and was employed for the 5′ UTR investigation of the *KLK* gene family members. Our results led to the accurate identification of the 5′ UTRs of the *BCL2L12* gene, being in accordance with the 5′ ends provided by both direct RNA and PCR-cDNA sequencing method from Oxford Nanopore Technologies. The implementation of the presented 5′ RACE-seq method not only confirmed the existence of annotated 5′ UTRs of the human *KLK* gene family members, but also led to the identification of novel and previously uncharacterized 5′ UTRs that are derived by complex splicing events. Besides the technical advancements, our approach provides a simple, cost-effective, and reliable workflow for the accurate 5′ UTR identification of any mRNA target.

## Methods

### Target gene selection

Our custom 5′ RACE-seq methodology was optimized on the apoptosis-related *BCL2L12* gene and was applied in members of the human *KLK* gene family. The human *KLK* mRNAs demonstrate notably differentiated expression levels in human tissues and at the same time they exhibit an extraordinary number of alternative splice variants. Nevertheless, their 5′ UTRs are not thoroughly identified and as a result they constitute candidate targets to study using novel 5′ RACE approaches. On the other hand, *BCL2L12* mRNA levels demonstrate a wider, yet more constant expression pattern in human tissues. In addition, since it’s 5′ UTRs have already been extensively studied, *BCL2L12* represents an ideal control gene for the evaluation of the results obtained by the presented 5′ RACE-seq approach.

### Human cell line culture and total RNA isolation

The current study was implemented using a wide established panel of human cancer cell lines that correspond to multiple primary tissues (Supplementary Table 2). Cell lines were purchased from the American Type Culture Collection (ATCC) and were propagated according to the guidelines. Total RNA isolation from each cell line was carried out with the TRIzol Reagent (Ambion™, Thermo Fisher Scientific Inc., Waltham, MA, USA). Purity and concentration of each total RNA sample was determined spectrophotometrically at 260 and 280 nm, using a BioSpec-nano Micro-volume UV–Vis Spectrophotometer (Shimadju, Kyoto, Japan).

### Template-switching oligonucleotide design

To generate full-length cDNA libraries compatible for downstream 5′ and/or 3′ RACE reactions, reverse transcription (RT) was carried out utilizing an oligo-dT-adapter as RT primer and a reverse transcriptase that has an associated terminal nucleotidyl transferase (TdT)-like activity, which enables the addition of non-templated nucleotides to the 3′ ends of DNA. In the present study, the SMARTScribe™ Reverse Transcriptase (Takara Bio, Inc) was used due to its enhanced terminal transferase activity. Accordingly, TSOs were designed to anneal at the extra added nucleotides, exploiting the 5′ cap-dependent template-switching mechanism. This methodology results to the production of full-length cDNAs, which incorporate totally defined 5′ and 3′ ends. The selection of the most appropriate TSO was conducted during optimization of the RT reaction, where three different TSOs were used (Table 1).

Besides the usage of an unmodified TSO that was composed of typical DNA bases, we designed two additional TSOs that contained a combination of monomeric units and specific modifications, thus were expected to increase the efficiency of the template-switching mechanism. In detail, a chimeric DNA/RNA TSO consisting of a DNA oligo sequence that carries 3 riboguanosines r(GGG) at its 3′ end (drTSO) was utilized, due to the fact that rG bases and the 3′ dC extension of the cDNA molecule enhances the subsequent template switching. Finally, the last designed TSO was the same chimeric DNA/RNA sequence, but also included a C3-spacer modification in the last nucleotide of the 3′ end (C3-TSO). This blocking strategy was employed to increase the specificity of 5′ RACE, since the use of unblocked TSOs might lead to non-specific cDNA products that will introduce bias to the approach.

### First-strand synthesis of full-length cDNAs

An initial amount of 2 μg total RNA from each human cell line was used as template for the first-strand cDNA synthesis, using an oligo-dT-adapter as RT primer, which was designed to anneal in the 3′ poly(A) tail of the mRNA transcripts. The nucleotide sequence of the oligo-dT-adapter was the following: 5′ - GCGAGCACAGAATTAATACGACTCACTATAGGTTTTTTTTTTTTVN - 3′ (where V = G, A, C and N = G, A, T, C). The reaction mixture for RNA denaturation contained 2 μl total RNA (2 μg), 1 μl oligo-dT-adapter (10 μM) and was adjusted with nuclease-free H_2_O to a final volume of 4.5 μl. The annealing of the oligo-dT-adapter primer to the poly(A) tail of mRNAs was performed by incubating the reaction mix for 3 min at 72 °C, followed by 2 min at 42 °C, in a Veriti 96-Well Fast Thermal Cycler (Applied Biosystems™). Thereafter, the reaction mix was immediately placed on ice until the downstream RT reaction. At this point, to optimize the RT reaction we conducted two distinct approaches.

In the first approach, we employed a typical 1-step RT reaction, where the designed TSOs were added at the beginning of the RT. The final cDNA synthesis reaction volume was adjusted to 10 μl, by adding the following reagents: 2 μl of 5X First-Strand Buffer, 0.25 μl DTT (100 mM), 1 μl dNTP mix (10 mM each), 1 μl TSO (10 μM), 0.25 μl RNaseOUT inhibitor (10 U) (Invitrogen™, Thermo Fisher Scientific Inc.) and 1 μl (100 U) SMARTScribe™ Reverse Transcriptase (Takara Bio, Inc). The final cDNA synthesis mix was incubated at 42° for 90 min, followed by a thermal inactivation of the RT enzyme at 72 °C for 10 min.

However, the timepoint where the TSO was added to the RT reaction mix is a critical parameter for the efficiency of the template-switching mechanism. Theoretically, the addition of the TSO at the beginning of the RT is expected to enhance the possibility of TSO mispriming events, thus leading to the synthesis of unwanted cDNA products. On the other hand, the addition of the TSO in a later step, after the complete synthesis of cDNAs, is expected to increase the efficiency of the 5′ RACE, since the only available template will be the extra nucleotides to the 3′ ends of DNA that are added by the RT enzyme.

**Fig. 6:**
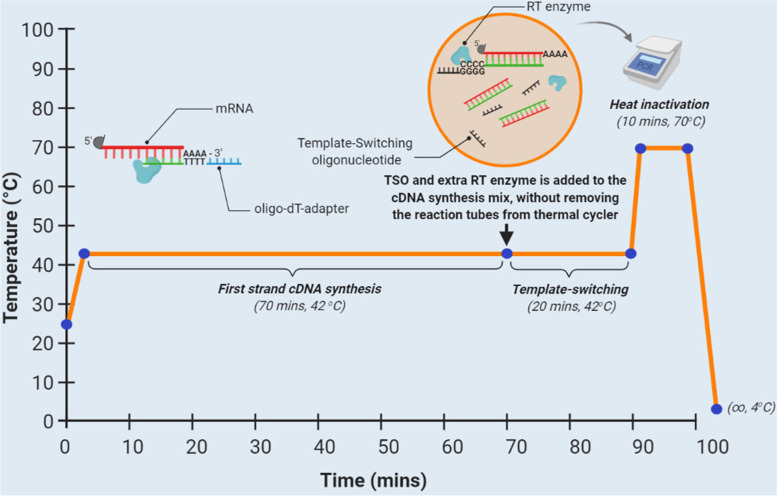
Schematic demonstration of the custom designed 2-step RT protocol presented in this study. The cDNA synthesis is performed using an oligo-dT-adapter as RT primer and SMARTScribe™ Reverse Transcriptase (Takara Bio, Inc), which has an enhanced terminal nucleotidyl transferase (TdT)-like activity. After the full-length cDNA synthesis by incubation of the reaction mix at 42° for 70 min, a template-switching oligonucleotide (TSO) is added to the reaction mix, without removing the tubes from the thermal cycler. The reaction mix containing the TSO is further incubated at 42° for additional 20 min to facilitate the template-switching mechanism. Finally, thermal inactivation of the RT enzyme is achieved by incubating the RT mixture at 72 °C for 10 min

Taking all these aspects under consideration, in the second approach, we developed a 2-step RT protocol, in which the addition of the TSO was carried out during a specific timepoint of the RT reaction, thus completely separating the cDNA extension process (1st Step) from the template-switching mechanism (2nd Step). In this case, the final cDNA synthesis mixture volume was adjusted to 10 μl, by adding the following reagents: 1.25 μl nuclease-free H_2_O, 2 μl of 5X First-Strand Buffer, 0.25 μl DTT (100 mM), 1 μl dNTP mix (10 mM each), 0.25 μl RNaseOUT inhibitor (10 U) (Invitrogen™, Thermo Fisher Scientific Inc.), 0.75 μl (75 U) SMARTScribe™ Reverse Transcriptase (Takara Bio, Inc). The reaction mix was incubated at 42° for 70 min to enable the cDNA synthesis. At this point, 1 μl TSO (10 μM) and 0.25 μl (25 U) SMARTScribe™ Reverse Transcriptase (Takara Bio, Inc) were added to the reaction mix, without removing the reaction tubes from the thermal cycler (Fig. 6). The reaction mix was then incubated at 42° for additional 20 min to facilitate the template-switching mechanism. Finally, the thermal inactivation of the RT enzyme was achieved by incubating the RT mixture at 72 °C for 10 min (Fig. 6). The quality control of the obtained cDNAs was performed with PCR amplification of the housekeeping gene Glyceraldehyde 3-phosphate dehydrogenase (*GAPDH*), using two GSP primers (Supplementary Table 1). The final cDNA products were diluted 1:5 and were pooled in a final cDNA mix, which was used as template for the downstream 5′ RACE reactions.

### Rapid amplification of cDNA 5′ ends

Amplification of 5′ cDNA ends was performed for *BCL2L12* and members of the *KLK* gene family, using a universal forward primer (F) designed to anneal at the TSO sequence and a reverse gene-specific primer (GSP) for each target gene. The derived PCR products were diluted 1:50 in nuclease-free H_2_O and used as templates for a nested 5′ RACE to further increase yield as well as both specificity and sensitivity for 5′ UTRs. To achieve this, we used a second universal forward primer along with an inner GSP. The final 5′ RACE products were purified using the NucleoSpin® Gel and PCR Clean-up kit (Macherey-Nagel GmbH & Co. KG, Duren, Germany) and electrophorized to assess the efficacy of the different RT protocols. The gene-specific primers for *BCL2L12* and *KLKs* are described in Supplementary Table 1. The purified PCR products from each target gene were pooled in a final product mix for the downstream library preparation workflow.

### 5′ RACE-seq with nanopore sequencing

An amount of 1 μg from the final PCR product mix was used as input for the library preparation workflow. Sequencing was performed in a MinION Mk1C system (Oxford Nanopore Technologies Ltd., ONT), using a FLO-MIN106 flow cell with R9.4.1 chemistry and the Ligation Sequencing Kit (SQK-LSK109, ONT) according to the manufacturer’s instructions. In particular, end repair process was carried out using the NEBNext® Ultra™ II End Repair/dA-Tailing Module (New England Biolabs, Inc), adapter ligation was carried out using Quick T4 Ligase (New England Biolabs, Inc), while purification between enzymatic reactions performed by AMPure XP magnetic beads (Beckman Coulter, USA).

### Bioinformatics analysis

The obtained FASTQ file containing the raw sequencing data was processed using Porechop to remove the library adapter sequences. The processed FASTQ file was aligned against the human reference genome (GRCh38) using Minimap2 [40]. In the next step, the Integrative Genomics Viewer (IGV) was used for the visualization of the successfully aligned reads and the assessment of the results [41]. The obtained results were compared with datasets derived from PCR-cDNA sequencing methodology (SQK-PCS109, ONT), which uses a template-switching mechanism to elucidate the full length of mRNAs. Finally, the experimental results of the presented 5′ RACE-seq methodology were compared to publicly available datasets derived from the Direct-RNA sequencing application, which is known to provide full-length mRNA transcripts with accurately defined ends, without the incorporation of template-switching at the library process.

## Supplementary Information

**Additional file 1: Supplementary Table 1.** List of gene-specific primers that were used in the present study. The melting temperature of each primer was calculated with Primer-BLAST designing tool.

**Additional file 2: Supplementary Figure 1.** Representative nanopore sequencing reads derived from the presented 5′ RACE-seq approach, which confirm the existence of the novel 5′ UTRs of the investigated *BCL2L12* and *KLK* genes. The nucleotides of each exon are exhibited in different colors for visual purposes.

**Additional file 3: Supplementary Figure 2.** Electrophoresis results of the nested 5′ RACE products regarding the human *BCL2L12* gene. The human cell lines of the present study were pooled based on the type of malignancy/tissue to generate distinct cDNA pools. The produced samples were used as templates for the implementation of the nested 5′ RACE.

**Additional file 4: Supplementary Figure 3.** Comparative analysis of aligned sequencing reads derived from the presented methodology and data provided by the FANTOM 5 consortium. The human genome GRCh38 was used as reference (blue color). The loaded BED file from FANTOM5 database showing the transcription start sites is shown in green color.

**Additional file 5: Supplementary Table 2.** The panel of 51 human cell lines that were used for the implementation of the present study. For each cell line the tissue of origin is also demonstrated.

**Additional file 6: Supplementary material (Original figures).**

**Additional file 7: ﻿Supplementary material (Original figures).**

## Data Availability

The dataset(s) supporting the conclusions of this article is(are) available in the Sequence Read Archive (SRA) repository, Submission ID: SUB10795831 - BioProject ID: PRJNA788520.

## Abbreviations

AS: Alternative splicing
ATCC: American Type Culture Collection
GSP: Gene-specific primer
IGV: Integrative Genomics Viewer
KLKs: Kallikrein-related peptidases
MMLV: Moloney murine leukemia virus
mRNA: messenger RNA
ncRNAs: non-coding RNAs
RACE: Rapid amplification of cDNA ends
RT: Reverse transcription
RNA-seq: RNA sequencing
SRA: Sequence Read Archive
TSO: Template-switching oligo
TdT: Terminal deoxynucleotidyl transferase
TAP: Tobacco acid pyrophosphatase
UTRs: Untranslated regions
