# Supplementary material for: A versatile 5′ RACE-Seq methodology for the accurate identification of the 5′ termini of mRNAs
Source: BMC Genomics. 2022 Feb 26;23:163. doi: 10.1186/s12864-022-08386-y (PMC8881849; doi:10.1186/s12864-022-08386-y)
Supplement: Supplementary file 1 — Additional file 1: Supplementary Table 1. List of gene-specific primers that were used in the present study. The melting temperature of each primer was calculated with Primer-BLAST designing tool. [file 12864_2022_8386_MOESM1_ESM.docx]

**Supplementary Table 1.** List of gene-specific primers that were used in the present study. The melting temperature of each primer was calculated with Primer-BLAST designing tool.

|  | **Gene-specific primers** | | | |
| --- | --- | --- | --- | --- |
| **Gene** | **PCR round** | **Sequence (5′→3′)** | **Length (nt)** | **T_m_ (^o^C)** |
| *KLK2* | 5' RACE | CAC ACC ATT ACA GAC AAG TGG AC | 23 | 59.50 |
|  | Nested | GAG TAA GCT CTA GCA CAC ATG TC | 23 | 58.89 |
| *KLK3* | 5' RACE | CTG TCC AGC GTC CAG CAC A | 19 | 62.52 |
|  | Nested | CAC ACA GCA TGA ACT TGG TCA C | 22 | 60.29 |
| *KLK5* | 5' RACE | AGT CTC TAC CTG CTT TGT CAC C | 22 | 59.70 |
|  | Nested | CAT GGT GTC ATC TAT CTG TCT CGG | 24 | 60.56 |
| *KLK7* | 5' RACE | CCC TGA GTC ACC ATT GCA GG | 20 | 60.68 |
|  | Nested | CTG TCG CCC AGC GTA TCA CT | 20 | 62.28 |
| *KLK8* | 5' RACE | GCC AGG TTT GTC GGA CCT C | 19 | 60.67 |
|  | Nested | TCC AGA ATC GCC CTG GCA | 18 | 61.01 |
| *KLK10* | 5' RACE | GAG TCA CTC TGG CAA GGG T | 19 | 58.94 |
|  | Nested | GGT CCA GTC CAG CAC ATA TCA | 21 | 59.51 |
| *KLK12* | 5' RACE | CCA GGA CAC CAG ACC TTG AA | 20 | 59.24 |
|  | Nested | CGA TGG AGA GGT TGA GGC AC | 20 | 60.46 |
| *BCL2L12* | 5' RACE | CGA AGG CGG CTC AGG AA | 17 | 59.35 |
|  | Nested | CAG CCT CAC CAC GCC TAA G | 19 | 60.45 |
| *GAPDH* | Regular PCR | CCA CAT CGC TCA GAC ACC AT | 20 | 60.11 |
|  |  | TGA CAA GCT TCC CGT TCT CA | 20 | 59.24 |
