# Supplementary figures and images for: A versatile 5′ RACE-Seq methodology for the accurate identification of the 5′ termini of mRNAs
Source: BMC Genomics. 2022 Feb 26;23:163. doi: 10.1186/s12864-022-08386-y (PMC8881849; doi:10.1186/s12864-022-08386-y)

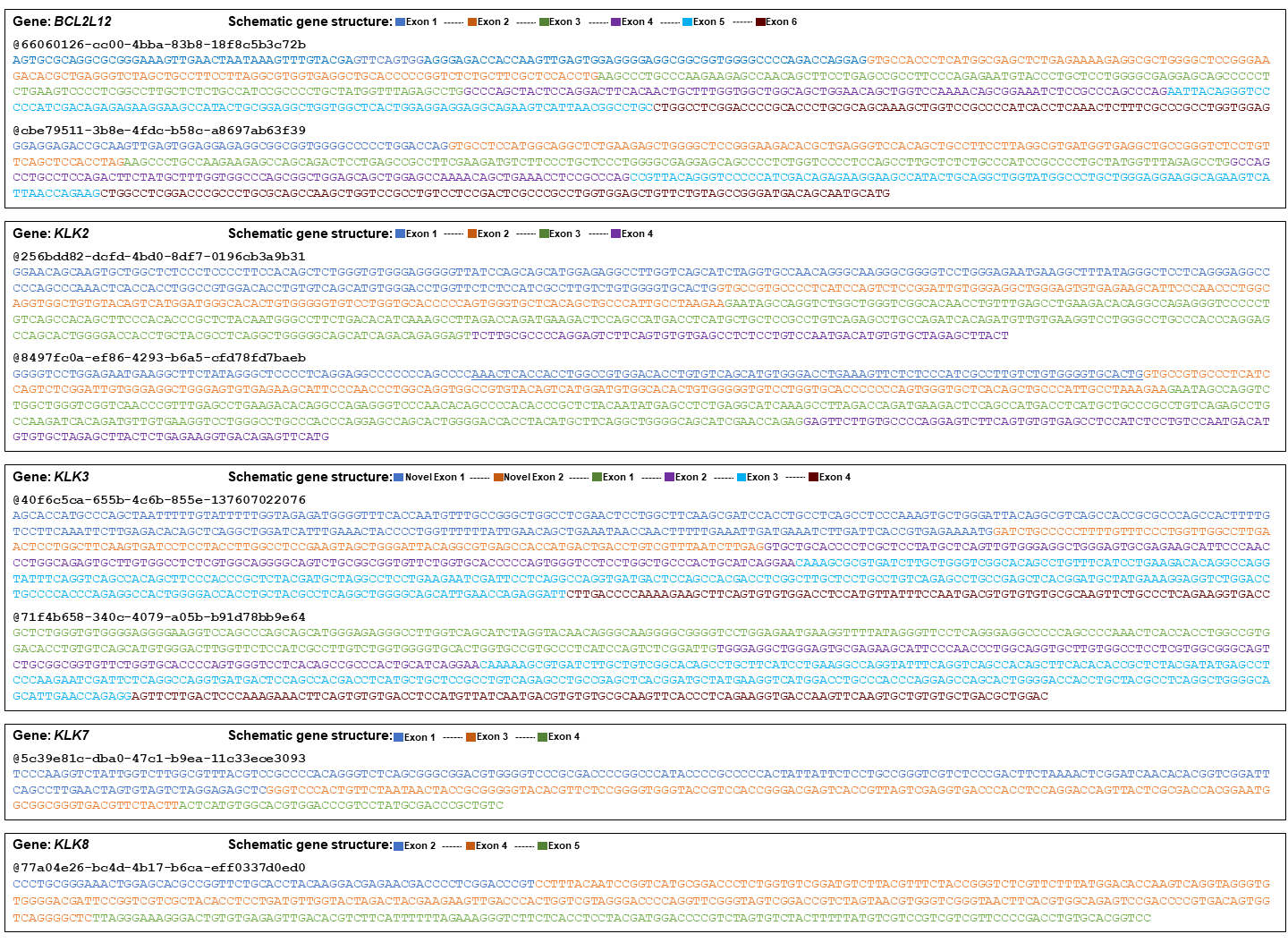

Supplement: Supplementary file 2 — Additional file 2: Supplementary Figure 1. Representative nanopore sequencing reads derived from the presented 5′ RACE-seq approach, which confirm the existence of the novel 5′ UTRs of the investigated BCL2L12 and KLK genes. The nucleotides of each exon are exhibited in different colors for visual purposes. [file 12864_2022_8386_MOESM2_ESM.tif]

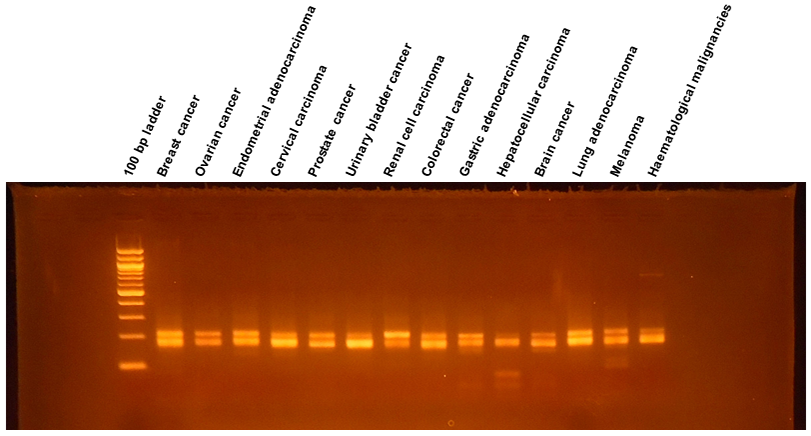

Supplement: Supplementary file 3 — Additional file 3: Supplementary Figure 2. Electrophoresis results of the nested 5′ RACE products regarding the human BCL2L12 gene. The human cell lines of the present study were pooled based on the type of malignancy/tissue to generate distinct cDNA pools. The produced samples were used as templates for the implementation of the nested 5′ RACE. [file 12864_2022_8386_MOESM3_ESM.tif]

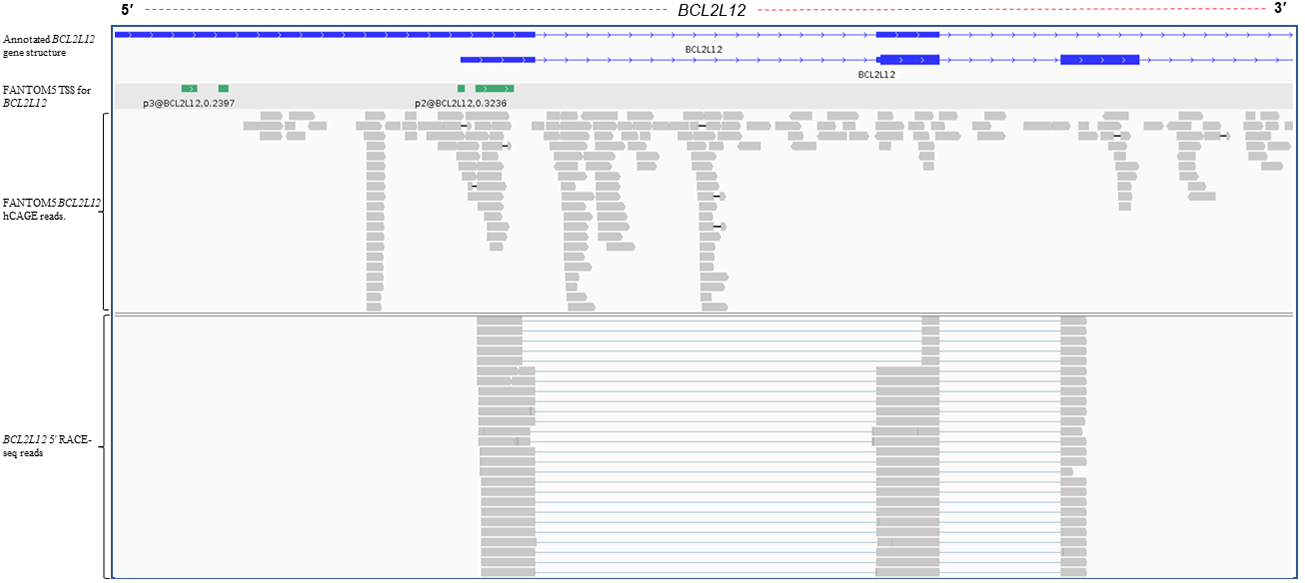

Supplement: Supplementary file 4 — Additional file 4: Supplementary Figure 3. Comparative analysis of aligned sequencing reads derived from the presented methodology and data provided by the FANTOM 5 consortium. The human genome GRCh38 was used as reference (blue color). The loaded BED file from FANTOM5 database showing the transcription start sites is shown in green color. [file 12864_2022_8386_MOESM4_ESM.tif]
