# Supplementary material for: A versatile 5′ RACE-Seq methodology for the accurate identification of the 5′ termini of mRNAs
Source: BMC Genomics. 2022 Feb 26;23:163. doi: 10.1186/s12864-022-08386-y (PMC8881849; doi:10.1186/s12864-022-08386-y)
Supplement: Supplementary file 5 — Additional file 5: Supplementary Table 2. The panel of 51 human cell lines that were used for the implementation of the present study. For each cell line the tissue of origin is also demonstrated. [file 12864_2022_8386_MOESM5_ESM.docx]

**Supplementary Table 2.** The panel of 51 human cell lines that were used for the implementation of the present study. For each cell line the tissue of origin is also demonstrated.

| **Human tissue of origin** | **Human Cell lines** |
| --- | --- |
| Breast cancer | MCF-7, SK-BR-3, BT-20, MDA-MB-231, MDA-MB-468, BT-474, T-47D, ZR-75-1 |
| Ovarian cancer | OVCAR-3, SK-OV-3, ES-2, MDAH-2774 |
| Endometrial adenocarcinoma | Ishikawa, SK-UT-1B |
| Cervical carcinoma | HeLa, SiHa |
| Prostate cancer | PC-3, DU 145, LNCaP |
| Urinary bladder cancer | T24, RT4 |
| Renal cell carcinoma | ACHN, 786-O, Caki-1 |
| Colorectal cancer | Caco-2, DLD-1, HT-29, HCT 116, SW 620, COLO 205, RKO |
| Gastric adenocarcinoma | AGS |
| Hepatocellular carcinoma | Hep G2, HuH-7 |
| Brain cancer | U-87 MG, U-251 MG, D54, H4, SH-SY5Y |
| Lung adenocarcinoma | A549 |
| Melanoma | FM3, MDA-MB-435S |
| Haematological malignancies | Raji, Daudi, U-937, K-562, HL-60, Jurkat, REC-1, SU-DHL-1, GRANTA-519 |

.
