# Supplementary material for: A versatile 5′ RACE-Seq methodology for the accurate identification of the 5′ termini of mRNAs
Source: BMC Genomics. 2022 Feb 26;23:163. doi: 10.1186/s12864-022-08386-y (PMC8881849; doi:10.1186/s12864-022-08386-y)
Supplement: Supplementary file 6 — Additional file 6: Supplementary material (Original figures). [file 12864_2022_8386_MOESM6_ESM.pdf]

<u>Original agarose gel picture</u>

Original agarose gel picture demonstrating the electrophoresis results of the amplicons derived from nested 5' RACE and the housekeeping gene amplification. **A.** Electrophoresis of nested 5' RACE amplicons corresponding to the 5' UTR of the human *BCL2L12* gene, using the three distinct TSOs described in the present study. Each TSO was incorporated both in a typical 1-step and a custom designed 2-step RT reactions before the implementation of the nested 5' RACE. **B.** Electrophoresis of the amplicons corresponding to the *GAPDH* mRNA amplification, which was used as housekeeping for the present work.

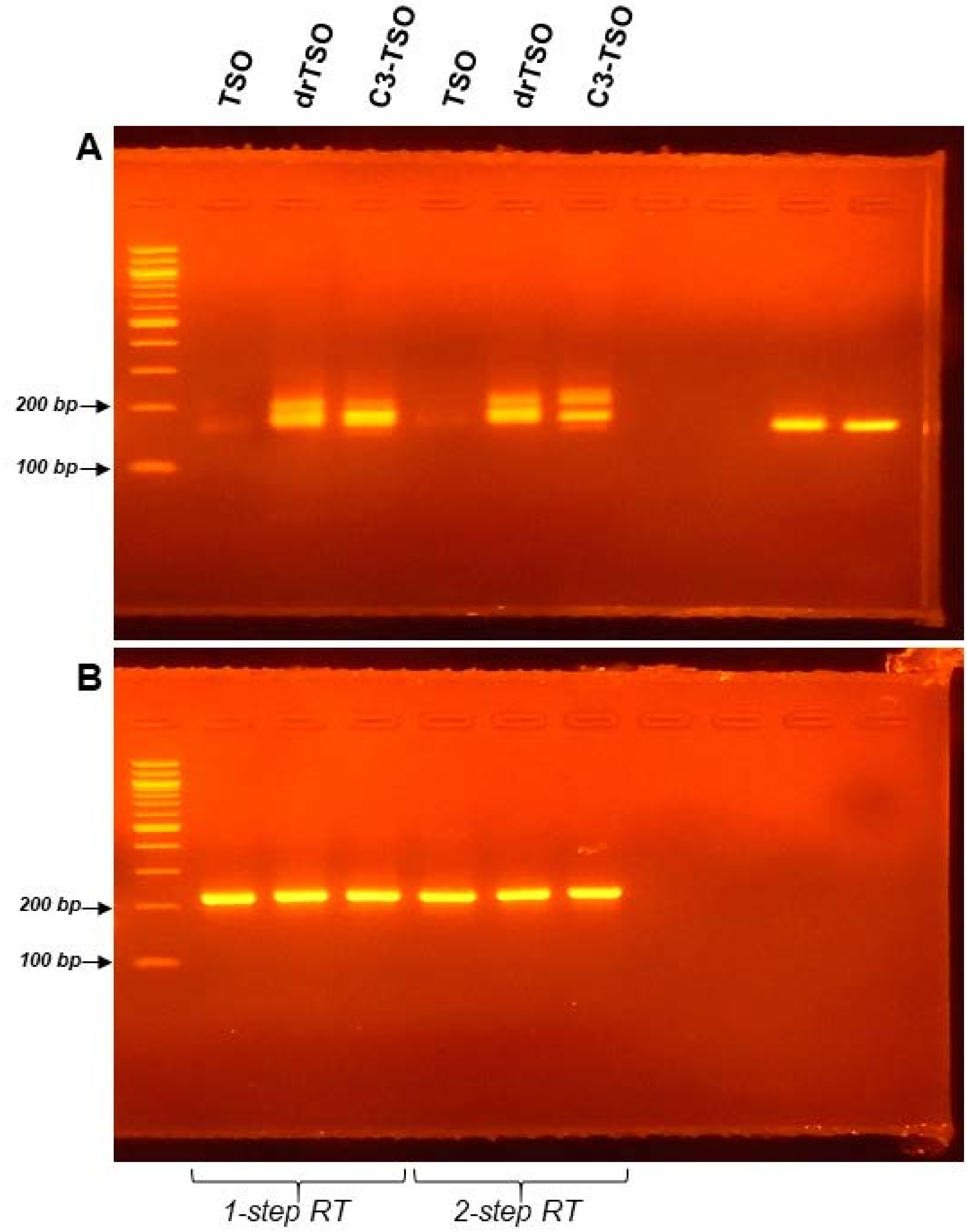
TSO
drTSO
C3-TSO
TSO
drTSO
C3-TSO
A
200 bp
100 bp
B
200 bp
100 bp
1-step RT
2-step RT
