# Supplementary material for: A versatile 5′ RACE-Seq methodology for the accurate identification of the 5′ termini of mRNAs
Source: BMC Genomics. 2022 Feb 26;23:163. doi: 10.1186/s12864-022-08386-y (PMC8881849; doi:10.1186/s12864-022-08386-y)
Supplement: Supplementary file 7 — Additional file 7: ﻿Supplementary material (Original figures). [file 12864_2022_8386_MOESM7_ESM.pdf]

Original IGV figures

Original IGV figures demonstrating the aligned sequencing reads derived from our custom designed 5' RACE-seq approach, which confirm the existence of annotated and novel UTRs for several members of the human *KLK* gene family. The human genome hg38 (GRCh38) was used as reference for the alignment process with minimap2.

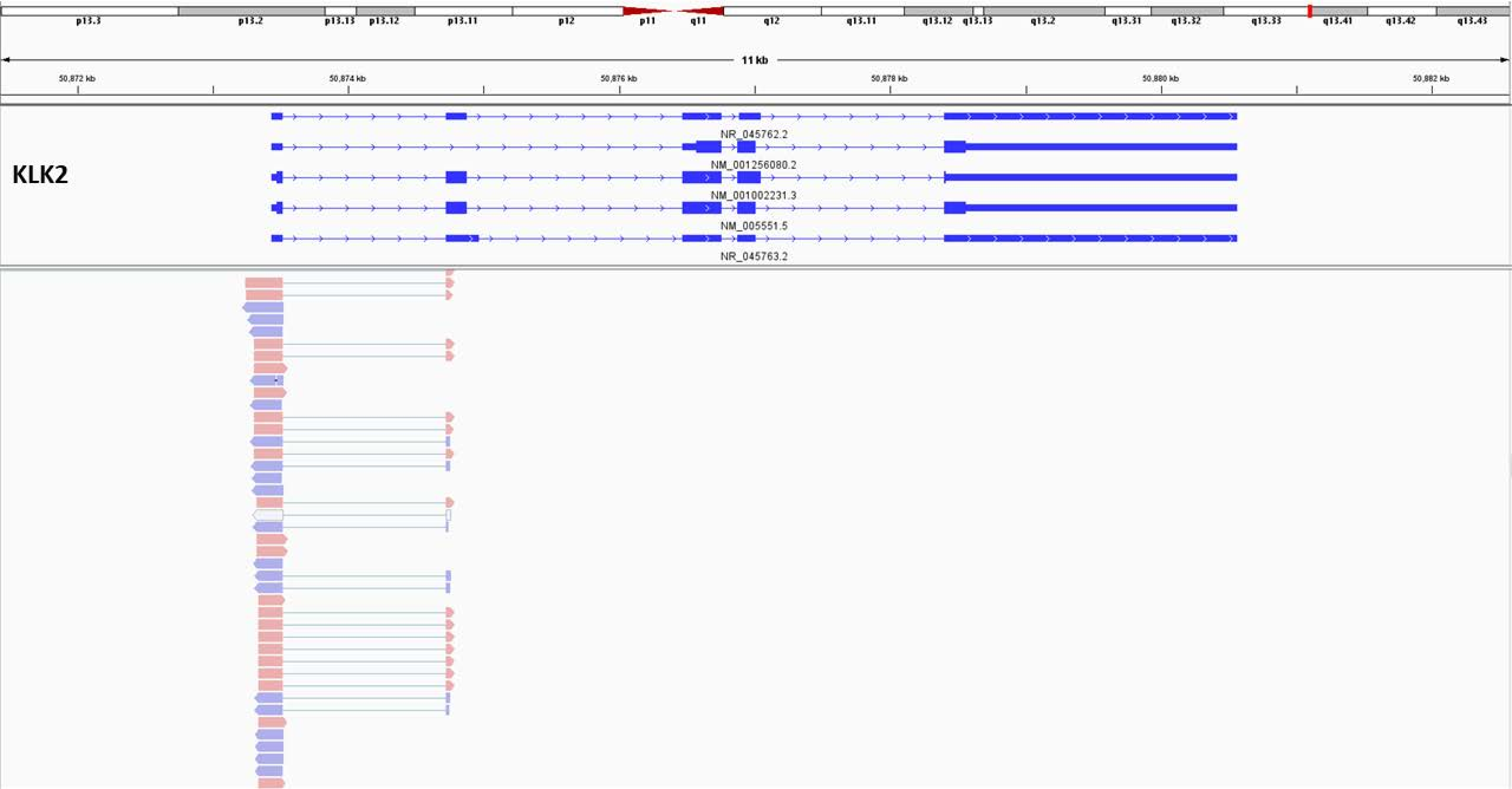
p13.3
p13.2
p13.13
p13.12
p13.11
p12
p11
q11
q12
q13.11
q13.12
q13.13
q13.2
q13.31
q13.32
q13.33
q13.41
q13.42
q13.43
11 kb
50,872 kb
50,874 kb
50,876 kb
50,878 kb
50,880 kb
50,882 kb
KLK2
NR_045762.2
NM_001256080.2
NM_001002231.3
NM_005551.5
NR_045763.2

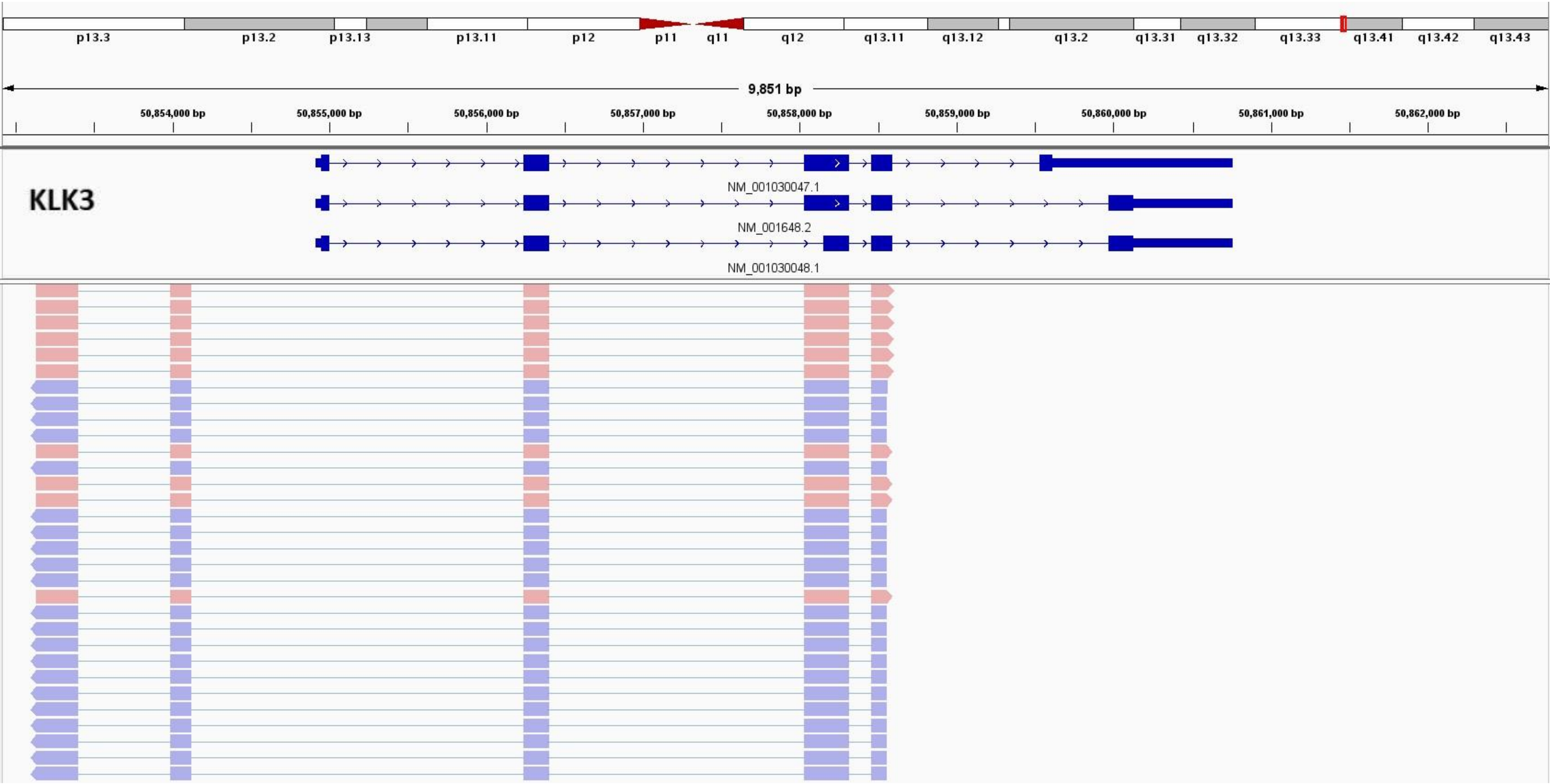
p13.3
p13.2
p13.13
p13.11
p12
p11
q11
q12
q13.11
q13.12
q13.2
q13.31
q13.32
q13.33
q13.41
q13.42
q13.43
9,851 bp
50,854,000 bp
50,855,000 bp
50,856,000 bp
50,857,000 bp
50,858,000 bp
50,859,000 bp
50,860,000 bp
50,861,000 bp
50,862,000 bp
KLK3
NM_001030047.1
NM_001648.2
NM_001030048.1

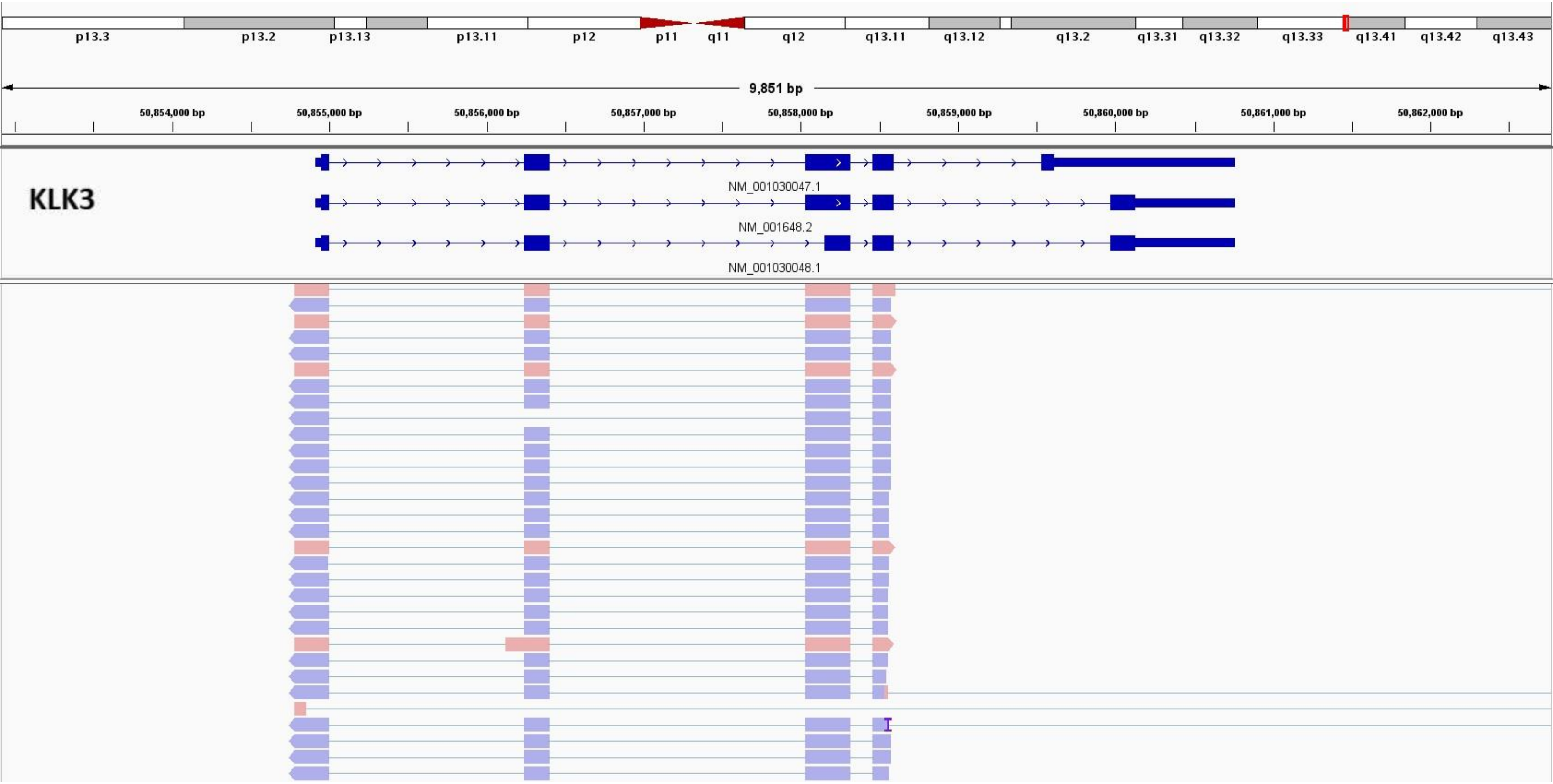
p13.3
p13.2
p13.13
p13.11
p12
p11
q11
q12
q13.11
q13.12
q13.2
q13.31
q13.32
q13.33
q13.41
q13.42
q13.43
9,851 bp
50,854,000 bp
50,855,000 bp
50,856,000 bp
50,857,000 bp
50,858,000 bp
50,859,000 bp
50,860,000 bp
50,861,000 bp
50,862,000 bp
KLK3
NM_001030047.1
NM_001648.2
NM_001030048.1

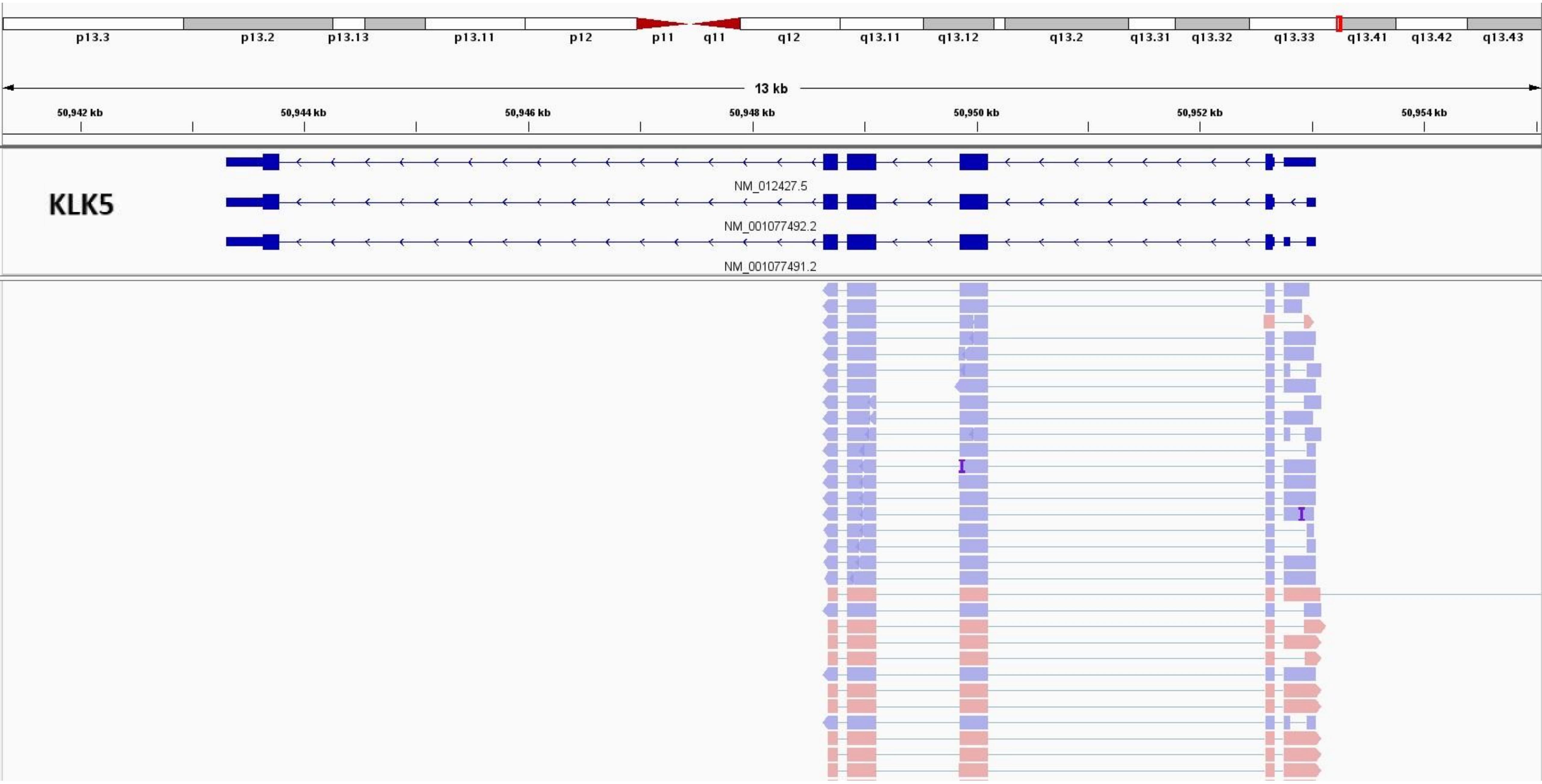
p13.3
p13.2
p13.13
p13.11
p12
p11
q11
q12
q13.11
q13.12
q13.2
q13.31
q13.32
q13.33
q13.41
q13.42
q13.43
13 kb
50,942 kb
50,944 kb
50,946 kb
50,948 kb
50,950 kb
50,952 kb
50,954 kb
KLK5
NM_012427.5
NM_001077492.2
NM_001077491.2

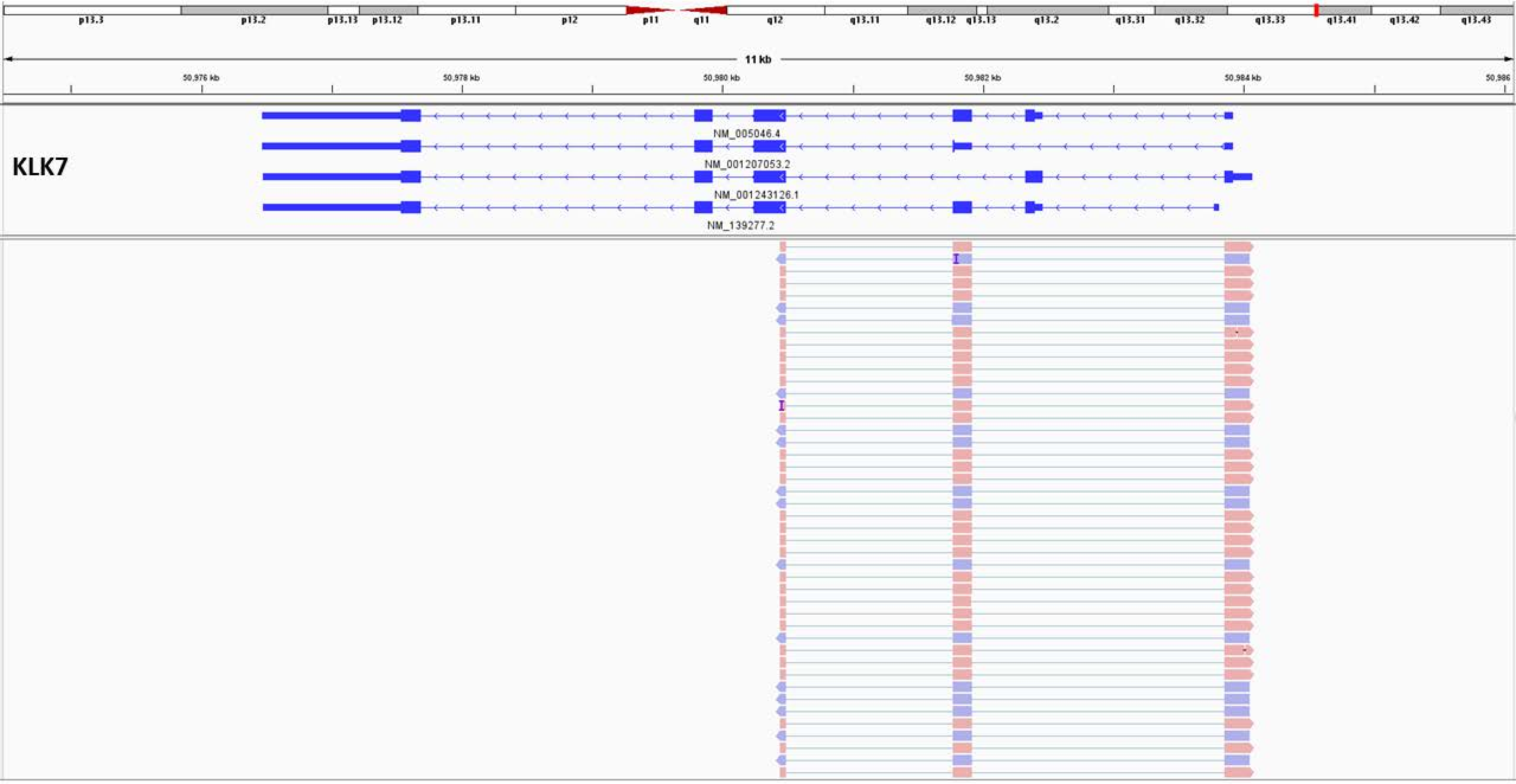

p13.3
p13.2
p13.13
p13.12
p13.11
p12
p11
q11
q12
q13.11
q13.12
q13.13
q13.2
q13.31
q13.32
q13.33
q13.41
q13.42
q13.43
11 kb
50,976 kb
50,978 kb
50,980 kb
50,982 kb
50,984 kb
50,986
KLK7
NM_005046.4
NM_001207053.2
NM_001243126.1
NM_139277.2

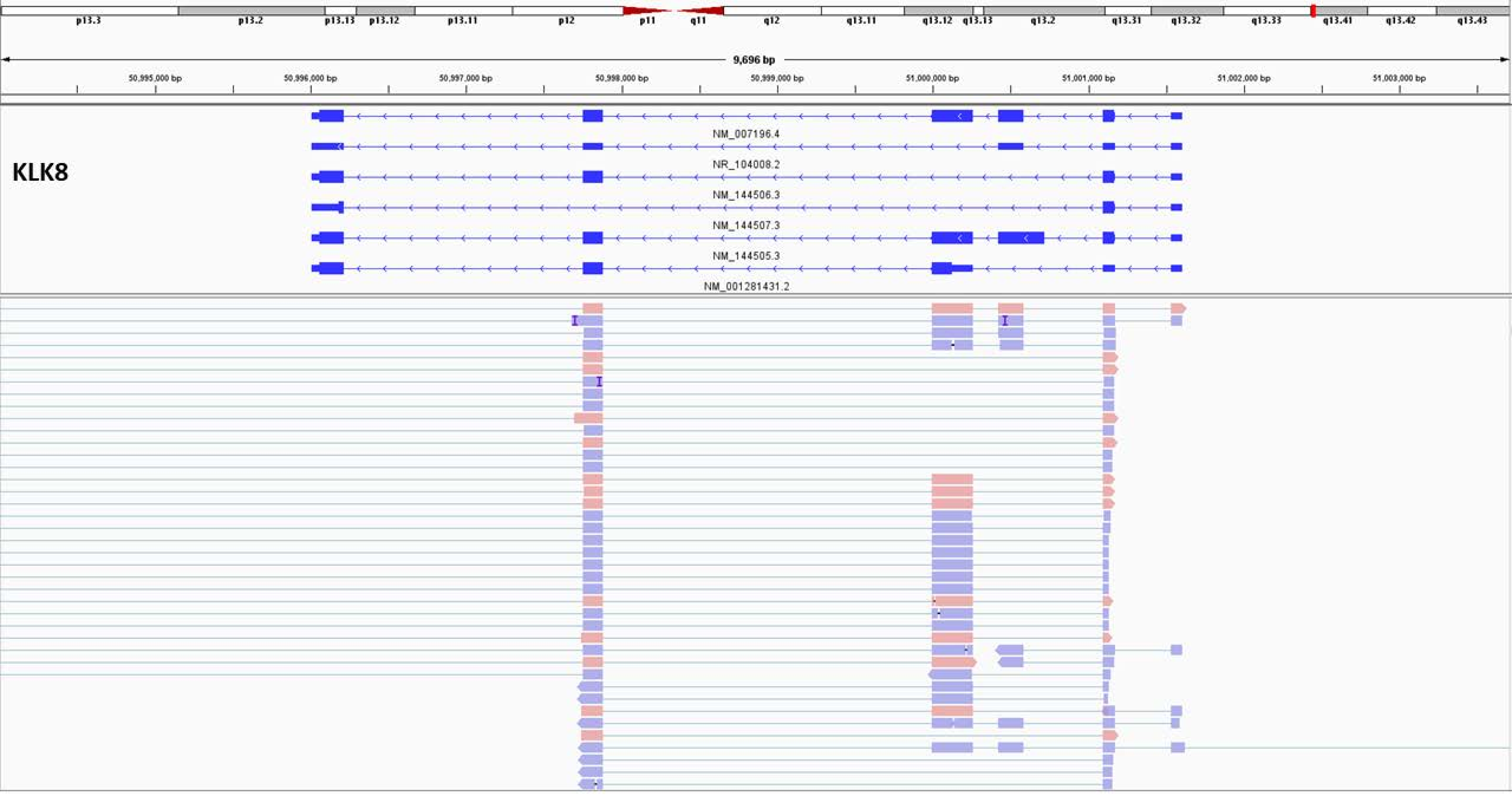

p13.3
p13.2
p13.13
p13.12
p13.11
p12
p11
q11
q12
q13.11
q13.12
q13.13
q13.2
q13.31
q13.32
q13.33
q13.41
q13.42
q13.43
9,696 bp
50,995,000 bp
50,996,000 bp
50,997,000 bp
50,998,000 bp
50,999,000 bp
51,000,000 bp
51,001,000 bp
51,002,000 bp
51,003,000 bp
KLK8
NM_007196.4
NR_104008.2
NM_144506.3
NM_144507.3
NM_144505.3
NM_001281431.2

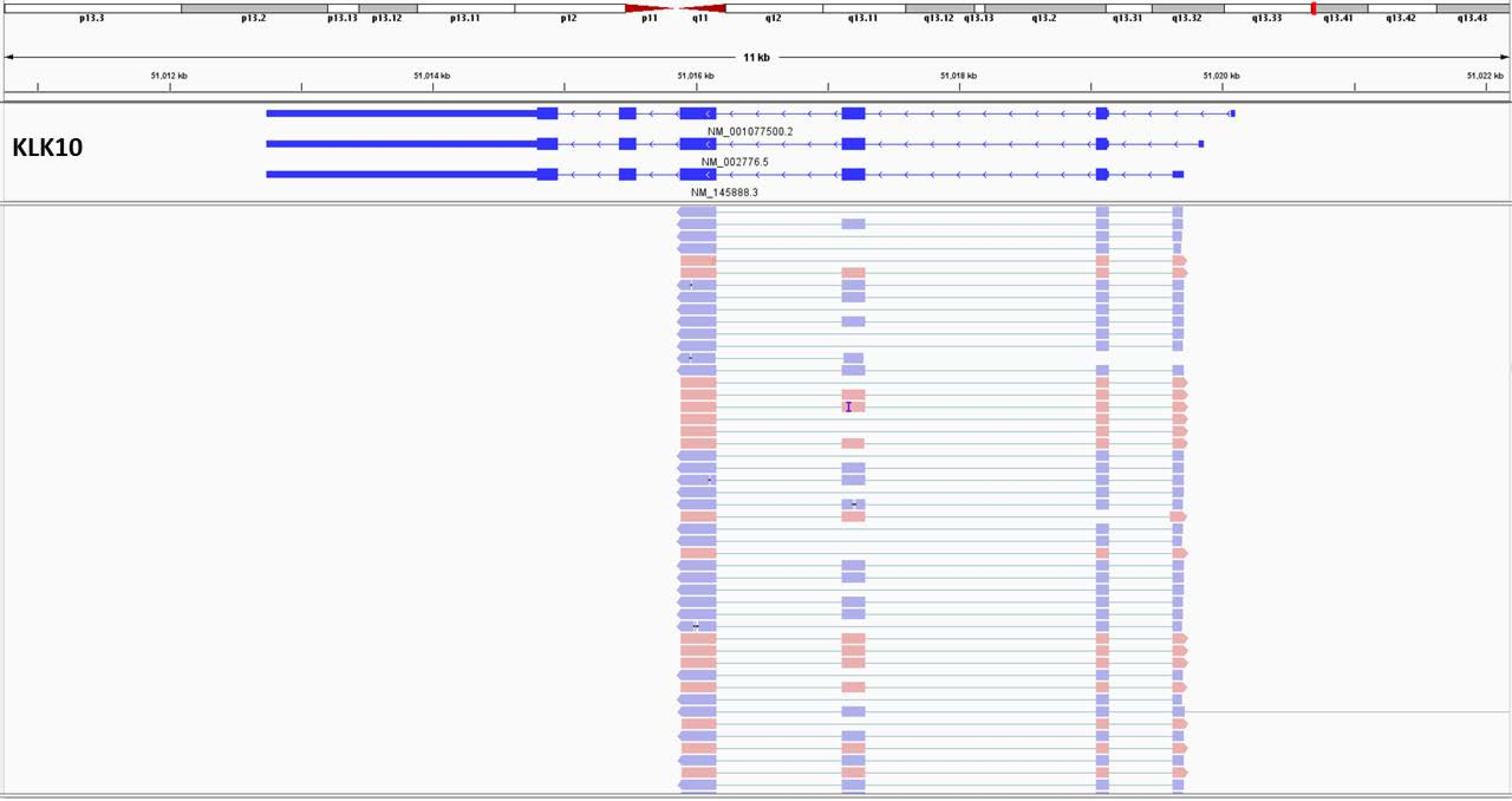
p13.3
p13.2
p13.13
p13.12
p13.11
p12
p11
q11
q12
q13.11
q13.12
q13.13
q13.2
q13.31
q13.32
q13.33
q13.41
q13.42
q13.43
11 kb
51,012 kb
51,014 kb
51,016 kb
51,018 kb
51,020 kb
51,022 kb
KLK10
NM_001077500.2
NM_002776.5
NM_145888.3

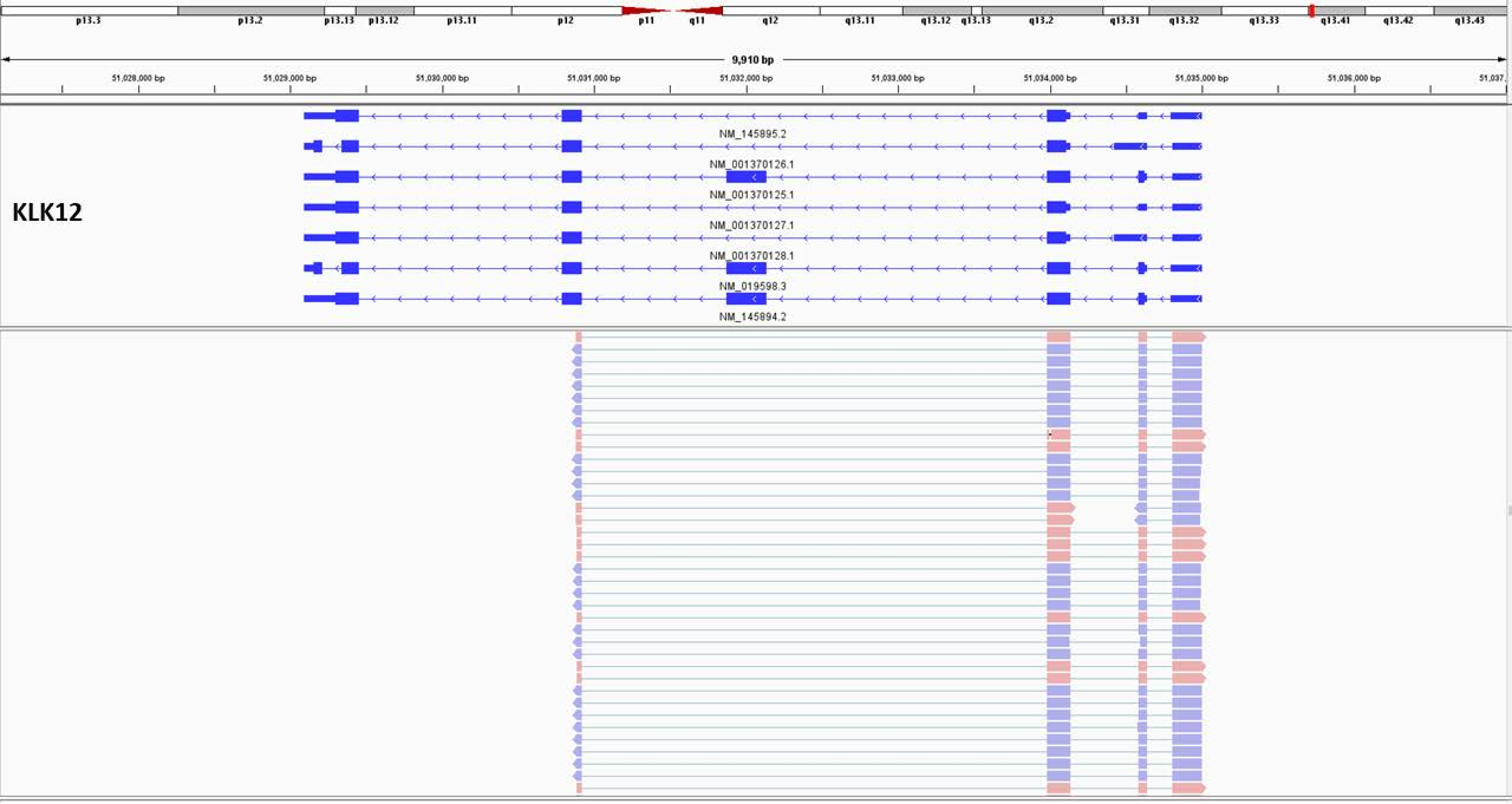

p13.3
p13.2
p13.13
p13.12
p13.11
p12
p11
q11
q12
q13.11
q13.12
q13.13
q13.2
q13.31
q13.32
q13.33
q13.41
q13.42
q13.43
9,910 bp
51,028,000 bp
51,029,000 bp
51,030,000 bp
51,031,000 bp
51,032,000 bp
51,033,000 bp
51,034,000 bp
51,035,000 bp
51,036,000 bp
KLK12
NM_145895.2
NM_001370126.1
NM_001370125.1
NM_001370127.1
NM_001370128.1
NM_019598.3
NM_145894.2
